# Awareness and practices survey of two ancient zoonotic diseases in Dhading District of Nepal

**DOI:** 10.1186/s12889-026-26757-y

**Published:** 2026-03-07

**Authors:** Michael Pudlo, Samagya Kharel, Hannah L. Welter, Rima D. Shrestha

**Affiliations:** 1 Department of Internal Medicine, University of Illinois College of Medicine Peoria (UICOMP), Peoria, IL USA; 2 Himalayan College of Agricultural Sciences and Technology (HICAST), Kalanki, Nepal; 3 Division of Research Services, Academic Affairs, University of Illinois College of Medicine Peoria (UICOMP), Peoria, IL USA

**Keywords:** Awareness, Practices, Rabies, Tuberculosis, TB, Zoonoses

## Abstract

**Background:**

Tuberculosis and rabies are two ancient zoonotic diseases that were discovered millennia ago, yet still claim thousands of lives worldwide in the twenty-first century. In Nepal, these diseases are endemic and pose a significant public health challenge in rural areas, such as the Dhading District, which has a low literacy rate and limited data availability. This study aims to address the gaps by evaluating knowledge of zoonotic diseases and identifying associated practices that can prevent their transmission.

**Methods:**

An in-person randomized cross-sectional survey was conducted in five Village Development Committees in the Dhading District using a standard, structured, and validated questionnaire. The participants were stratified into five groups based on their daily work and were expected to interview 50 people from each category per VDC. Questions related to these two diseases were used to determine knowledge scores and awareness. Descriptive statistics were calculated, and regression and mediation analysis were used to determine the factors that influence disease awareness and prevention practices.

**Results:**

This study interviewed 749 participants from five VDCs and achieved acceptable internal consistency and reliability (α = 0.74). Awareness of rabies was higher than that of TB (excellent: 2% vs 1%, and sufficient: 60% vs 11%) among these participants. Youth and women participants had significantly lower awareness of rabies and TB (p < 0.05). Mediation and path analysis indicated that TB knowledge is shaped more by structural and demographic variables rather than practice alone. In contrast, the rabies model highlights that while occupation strongly influences practices, its contribution to knowledge is complex and mediated by factors such as age, gender, and locality.

**Conclusions:**

Our findings emphasize that knowledge pathways differ across diseases even within the same population, necessitating tailored interventions. For TB, integrating education into occupational and practice-based platforms is critical, while for rabies, interventions should focus on overcoming gender and community-level disparities. Developing educational initiatives on zoonotic diseases and awareness among the public, specifically targeting women and youth, would benefit in reducing the burden of rabies and tuberculosis diseases in Nepal.

**Supplementary Information:**

The online version contains supplementary material available at 10.1186/s12889-026-26757-y.

## Background

Tuberculosis (TB) and rabies are two ancient zoonotic diseases, originating between 9000 and 4000 BCE, and have taken millions of lives worldwide [1–3]. Zoonotic diseases are those that can be transmitted between animals and humans. TB is a bacterial illness caused by *Mycobacterium tuberculosis* and manifests in humans as a granulomatous lung disease that most commonly causes symptoms such as cough, fatigue, and night sweats [4, 5]. TB is particularly dangerous because it can remain airborne in the respiratory droplets of an infected individual, allowing it to spread through the simple act of inhalation. Currently, the Bacille Calmette-Guérin (BCG) vaccine against mycobacterial diseases is available primarily for children in countries where TB is endemic to protect against severe forms of the disease [4]. If infected, treatment for TB involves a four-drug antibiotic therapy taken over 3 to 9 months, depending on the severity of the disease, as well as whether the infection is active or inactive [4]. Animal tuberculosis caused by *Mycobacterium bovis* in humans can be derived from risk factors, including cattle-related exposure through ingestion of raw dairy products, vocational exposure to livestock, and through consumption of unprocessed meat [6].

In contrast, rabies is a viral illness caused by the virus *Rabies lyssavirus*, and presents with delirium, fatigue, and hydrophobia in humans [7, 8]. Rabies is significantly more prevalent in animals such as dogs, raccoons, bats, and other wild animals, and characteristically presents with foaming at the mouth, aggression, and pica (an abnormal eating pattern that leads to consumption of things not considered food) [7–9]. Rabies is spread to humans primarily through animal bites and scratches. Vaccines are available for humans and animals; however, the vaccine's efficacy is low and requires booster shots periodically [10]. If infected, the only treatment available for humans involves wound care, a post-exposure prophylaxis vaccine, and human rabies immunoglobulin (HRIG). The vaccine and HRIG help the body immediately launch an immune response; however, treatment is effective only if given before the onset of physical symptoms [7].

In Nepal, a large proportion of the population (> 66%) is engaged in agricultural and animal husbandry across three terrains: mountainous regions where yak and sheep are bred, hilly areas where cows, sheep, goats, and poultry graze, and the Terai plains, which are home to buffalo, cows, goats, and poultry [2, 11]. The country faces high rates of tuberculosis (TB), especially in the Terai, hilly, and urban regions, driven by poor living conditions, multidrug-resistant strains, and the HIV/AIDS epidemic [11–14]. In 2021, TB affected 129 per 100,000 people, with 37.2% of pulmonary TB cases being multidrug-resistant, disproportionately impacting women [12]. The exact prevalence of bovine TB is underreported due to the lack of a coordinated national surveillance system in human and animal health services. In addition, there is a lack of diagnostic tools for differentiating the etiology of TB in humans from humans or cattle, leading to poor treatment outcomes [15]. Rabies is also remarkably endemic in Nepal, with 10 to 30 animal outbreaks monthly and fewer than 100 human rabies cases annually [8]. A large stray dog population has also contributed significantly to rabies transmission, with 96% of human rabies deaths linked to canine exposure [9]. Children and pedestrians are particularly vulnerable to bites from stray dogs [3, 8].

Limited resources in countries like Nepal could be significantly contributing to the continued endemicity of TB and rabies in the country [16]. These countries are especially at risk due to their predominance of a traditional agricultural society, limited resources for disease diagnosis and vaccinations or treatment, and a poor understanding of the disease and its transmission [16]. Additionally, natural calamities such as earthquakes or landslides severely affected the control of public health interventions for endemic diseases, leading to the transmission of infectious diseases. Thus, we chose to conduct this study in Dhading district, which had a severe impact of the 2015 massive earthquake in Nepal, had a low rate, poor health indicators, ~ 10 reported outbreaks of rabies annually, > 100,000 known TB cases, and a high percentage of poor and marginalized ethnic populations, such as the Chepang people, who traditionally eat bats [1, 14, 16–18].

In 1976, Koster introduced a Knowledge-Attitude-Practice (KAP) model for health behavior management in medical practice, disease prevention, and public health intervention. Knowledge indicates the public awareness of the topic, whereas attitude is belief, and practice is the action that could be changed if one intervenes in knowledge and attitude. The KAP has been used in Nepal and abroad for various diseases [19–21]. However, this was the first systematically conducted large study from the study area. In this study, we aim to identify awareness of zoonotic diseases in Village Development Committees (VDC) in the Dhading District of Nepal and to determine if profession, gender, and age affect the degree of knowledge of TB and rabies, and practices that affect the transmission of these two diseases. We predict that a reduced understanding of the disease process of zoonotic illnesses contributes to the persistence of TB and rabies in Nepal. To target efforts to reduce disease burden, identifying the degree of knowledge, attitudes, and practices within various demographics within the Dhading district is needed. Specifically, we identify the highest-risk groups that have the lowest understanding of the zoonoses of TB and rabies.

## Methods

### Study design, location, and population

**Fig. 1:**
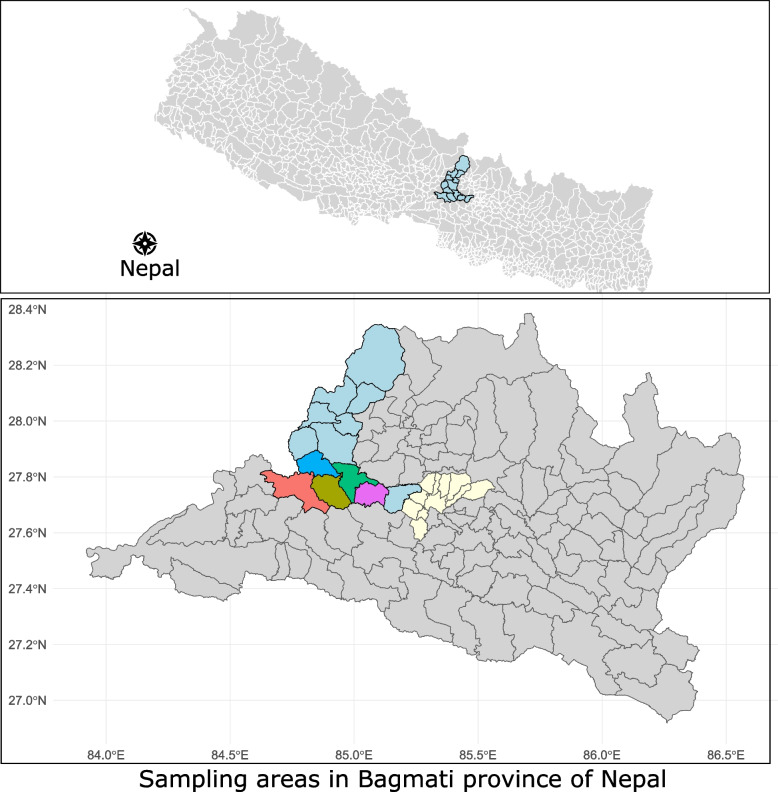
Sampling areas in Nepal (upper) for this study. The figure below shows Province 1, with Kathmandu district in yellow and Dhading district in blue. The other five colors represent the areas where participants were interviewed in the district

A cross-sectional study was conducted between February and April 2017 in five VDCs of Dhading District, Nepal (Fig. 1). Dhading district is a mountainous district with three ecozones in the Bagmati zone that is neighboring Kathmandu district. This district has diverse flora and fauna, along with integrated mixed methods of farming adopted by the farmers. The 2014 census reports showed 73,851 households with 336,067 people living in the Dhading district [2]. There were 181,620 goats, 363,473 cattle and buffaloes, and 314 metric tons of meat produced in the fiscal year 2015–2016 [22]. However, the district was harshly affected during the April 2015 earthquake [18]. Reports disclosed the collapse of more than 81,000 houses and thousands of animals. Natural disasters such as earthquakes and flood can lead to infectious disease threats and outbreaks, including those of zoonotic origin. Hence, we selected participants from five villages in the Dhading District.

To achieve the objective of this study, we purposely and randomly stratified the population of the villages, incorporating full-time jobs that they do daily (referred as occupation). These include students, teachers, farmers, butchers, and workers in animal and human health. Students represent a younger demographic, making them an ideal target for awareness and behavior change interventions [23–25]. Conversely, teachers play a critical role in disseminating knowledge and shaping attitudes within schools and communities [23, 25]. Farmers were included as they are highly relevant to the study objectives, given their occupational exposure. Animal health workers include anyone involved in treating animals, such as veterinary technicians and individuals holding community animal health certificates in agricultural medicine shops. Human health workers include anyone involved in treating people in those villages, such as pharmacists, nurses, community health workers, and doctors. These health workers each play a vital role in disease prevention, and identifying their awareness of zoonotic diseases is crucial for overall community health.

In addition, we stratify by age, allowing us to achieve the KAP from diverse age groups. For each category, at least 50 people were expected to be surveyed. All the participants residing in the five villages of Dhading who consented and completed the questionnaire were included in this study.

### Questionnaire development

We developed a questionnaire using the previously published questions and modified them according to Nepal’s five zoonotic disease scenarios. The questionnaire includes demographic information as well as open-ended and closed-ended questions on rabies and tuberculosis diseases, which reflect the participants' knowledge and practices (Additional file 1: Survey Questionnaire). Before implementing the final questionnaire, survey instruments were validated with experts and 10 participants, 2 from each occupation. The validity of the questionnaire was assessed through the experts' opinions and by matching their responses with the pre-survey participants' responses to these questions. The final questionnaire consisted of three dimensions: basic information, knowledge questions, and practice information. Seven questions were included to assess the knowledge of each disease, and eight questions related to practices that can impact exposure to tuberculosis or rabies. Zoonotic tuberculosis (TB) is indicated as bovine TB, but we asked questions about both TB to obtain participants' awareness to determine if they can understand the zoonotic transmission potential for either of them.

The research was carried out in accordance with the Declaration of Helsinki. During the study period, formal ethical review board approval from the National Health Research Council (NHRC) was not required for veterinary research or theses. However, administrative permission was obtained from the institutional research committee prior to conducting the study. This permission does not constitute national-level ethical approval. All adult participants were informed, and written consent was obtained before conducting the survey. Similarly, informed consent for students (< 18 years old) was obtained from all the school authorities, schoolteachers, and parents, and confidentiality was maintained. A survey of these students was conducted in the presence of the school's teachers. All participants were guaranteed anonymity and the right to terminate the interview at any time, in accordance with protocols on the rights of human subjects in research.

### Data collection and analysis

Each person was given a unique identification number, and the data collected on paper was stored in the Microsoft Excel 2007 program. These data were checked and validated before being analyzed in R software version 4.3 [26] using the RStudio environment [27]. One point is given for each correct answer to the disease-related knowledge questions. The maximum score for the knowledge was 15 for each disease. The total scores obtained were normalized using the formula:

$$X_{\mathrm{norm}}=\left(X-X_{\mathrm{min}}\right)/\left(X_{\mathrm{max}}-X_{\mathrm{min}}\right)$$

The normalized total scores are the knowledge score for that disease. These scores were used to determine awareness and are categorized as “Excellent”, “Good”, “Sufficient,” and “Poor” if the knowledge score is > 90%, > 75–90%, > 50–75%, and < 50%, respectively. Similar to knowledge, questions related to practice associated with the two diseases were scored and categorized as “Excellent”, “Good”, “Sufficient,” and “Poor” practices if the score is > 90%, > 75–90%, > 50–75%, and < 50%, respectively. The maximum score for practices was 14 and 11 for tuberculosis and rabies, respectively.

Chi-square or Fisher’s exact test was used for categorical group comparison of data that are expressed as a number (%). Mean, median, standard deviation (SD), and range were calculated for continuous data, and ANOVA was used for group comparison. To examine the correlation between knowledge and practice, the Pearson correlation was calculated. To explore the relationships among categorical variables (demographic variables vs awareness levels), multiple correspondence analysis (MCA) was employed. MCA helps to visualize patterns among categorical variables and identify similar clusters [28]. Univariable and multivariable regression analyses were also performed to determine the factors influencing/affecting the awareness of each disease. In addition, we applied mediation analysis to estimate the causality among knowledge, practices, and intermediate factors and visualize the path. This technique helps reveal whether improving knowledge indirectly leads to better health practices by affecting attitudes or other behaviors [29]. A *p-*value (p) of less than 0.05 was considered statistically significant.

## Results

### Demographic characteristics

A total of 746 residents of Dhading participated in the survey. Of them, 425 (57.0%) identified themselves as female (Table 1). The mean age of participants was 30 (SD: 10) years. The largest proportion of participants belonged to the 26–45-year age range (*n* = 267, 35.80%), and the lowest proportion was in the > 45-year age range (*n* = 129, 17.30%). Most of the female participants (*n* = 142, 33.4%) were < 18 years old, whereas 44.9% (n = 144) of the males were in the 26–45-year age range. The majority of participants were residing in VDC-2 (*n* = 172, 23.10%) and VDC-1 (*n* = 170, 22.80%), and the lowest number of participants was in VDC-4 (*n* = 81, 10.90%). Students made up the largest proportion of participants (*n* = 255, 34.10%), followed by farmers (*n* = 215, 28.80%). Human healthcare workers (*n* = 38, 5.10%) and others (*n* = 31, 4.20%), including meat-shop owners and animal health workers, also participated in this study.

**Table 1:** Demographic characteristics of Dhading District distributed by occupation of the surveyed population. The group labeled as “Others” includes animal health workers and meat shop workers

Variables	Health Worker	Teacher	Farmer	Student	Others	Total	*P* value
Number of Participants	38 (5.1%)	207 (27.7%)	215 (28.8%)	255 (34.2%)	31 (4.2%)	746	< 0.001
Age							
Mean (SD)	33 (11.0)	33 (10.8)	40 (10.8)	16 (2.1)	36 (8.8)	29 (13.2)	
Median (q1, q3)	30 (25.2, 39.2)	30 (25.0, 41.0)	38 (32.0, 47.0)	16 (14.5, 17.0)	37 (28.0, 42.0)	27 (17.0, 38.0)	
Range	20—59	18—60	20—81	12—32	22—54	12—81	
Age Group							< 0.001
< 18	0 (0.0%)	0 (0.0%)	0 (0.0%)	209 (82.0%)	0 (0.0%)	209 (28.0%)	
18–25	10 (26.3%)	59 (28.5%)	22 (10.2%)	45 (17.6%)	5 (16.1%)	141 (18.9%)	
26–45	22 (57.9%)	105 (50.7%)	119 (55.3%)	1 (0.4%)	20 (64.5%)	267 (35.8%)	
> 55	6 (15.8%)	43 (20.8%)	74 (34.4%)	0 (0.0%)	6 (19.4%)	129 (17.3%)	
Gender							
Male	14 (36.8%)	112 (54.1%)	95 (44.2%)	80 (31.4%)	20 (64.5%)	321 (43.0%)	< 0.001
Female	24 (63.2%)	95 (45.9%)	120 (55.8%)	175 (68.6%)	11 (35.5%)	425 (57.0%)	
VDC							
1	8 (21.1%)	49 (23.7%)	51 (23.7%)	50 (19.6%)	3 (9.7%)	161 (21.6%)	< 0.001
2	10 (26.3%)	50 (24.2%)	50 (23.3%)	50 (19.6%)	12 (38.7%)	172 (23.1%)	
3	15 (39.5%)	43 (20.8%)	53 (24.7%)	52 (20.4%)	7 (22.6%)	170 (22.8%)	
4	2 (5.3%)	14 (6.8%)	11 (5.1%)	53 (20.8%)	1 (3.2%)	81 (10.9%)	
5	3 (7.9%)	51 (24.6%)	50 (23.3%)	50 (19.6%)	8 (25.8%)	162 (21.7%)	

### Reliability and validity of the survey

The overall Feldt’s and Duhachek’s Cronbach’s alpha for this study is 0.74 [95% CI: 0.72–0.77], indicating good internal consistency and reliability of the survey results used in this study. Barlett’s test of sphericity is excellent (*p* = < 0.0001) for tuberculosis but not for rabies (*p* = > 0.05) survey.

### Knowledge/awareness of tuberculosis and rabies

The overall mean knowledge score of participants was 4.98 (± 2.21) and 9.49 (± 3.22) for tuberculosis and rabies, respectively. Tables 2 and 3 show the distribution of standardized knowledge scores for tuberculosis and rabies by occupation, VDC, gender, and age groups.

**Table 2:** Knowledge/awareness of tuberculosis and associated practices that can affect its transmission

Variables	Health worker (*N* = 38)	Farmer (*N* = 215)	Teacher (*N* = 207)	Student (*N* = 255)	Others (*N* = 31)	Total (*N* = 746)	*P* value
Total Knowledge Score							< 0.001
Mean (SD)	9 (2.8)	6 (1.6)	6 (2.0)	3 (1.2)	6 (2.5)	5 (2.2)	
Median (q1, q3)	9 (6.2, 11.0)	6 (4.5, 7.0)	6 (4.0, 7.0)	3 (3.0, 4.0)	6 (4.0, 7.5)	5 (3.0, 6.0)	
Range	3.0—13.0	1.0—11.0	2.0—12.0	1.0—7.0	1.0—12.0	1.0—13.0	
Standardized Knowledge Score							< 0.001
Mean (SD)	64 (23.7)	38 (13.6)	39 (16.5)	19 (9.6)	42 (20.8)	33 (18.4)	
Median (q1, q3)	67 (43.7, 83.2)	42 (29.1, 50.0)	42 (25.0, 50.0)	17 (16.6, 25.0)	42 (25.0, 54.1)	33 (16.6, 41.6)	
Range	16.6—100.0	0.0—83.2	8.2—91.6	0.0—50.0	0.0—91.6	0.0—100.0	
Awareness							< 0.001
Excellent	8 (21.1%)	0 (0.0%)	1 (0.5%)	0 (0.0%)	1 (3.2%)	10 (1.3%)	
Good	4 (10.5%)	1 (0.5%)	1 (0.5%)	0 (0.0%)	1 (3.2%)	7 (0.9%)	
Poor	13 (34.2%)	195 (90.7%)	169 (81.6%)	255 (100.0%)	23 (74.2%)	655 (87.8%)	
Sufficient	13 (34.2%)	19 (8.8%)	36 (17.4%)	0 (0.0%)	6 (19.4%)	74 (9.9%)	
Total Practice Score							< 0.001
Mean (SD)	7 (2.4)	8 (1.7)	7 (2.4)	9 (1.8)	7 (2.1)	8 (2.1)	
Median (q1, q3)	8 (5.0, 9.0)	8 (8.0, 9.0)	8 (5.0, 9.0)	9 (8.0, 9.0)	8 (4.0, 8.5)	8 (7.0, 9.0)	
Range	4.0—12.0	3.0—11.0	3.0—13.0	3.0—13.0	4.0—9.0	3.0—13.0	
Standardized Practice Score							< 0.001
Mean (SD)	41 (24.2)	48 (17.1)	40 (23.8)	55 (17.9)	39.0 (21.0)	48 (21.0)	
Median (q1, q3)	45 (20.0, 60.0)	50 (50.0, 60.0)	50 (20.0, 60.0)	60 (50.0, 60.0)	50 (10.0, 55.0)	50 (40.0, 60.0)	
Range	10.0—90.0	0.0—80.0	0.0—100.0	0.0—100.0	10.0—60.0	0.0—100.0	
Standardized Practices							< 0.001
Excellent	0 (0.0%)	0 (0.0%)	2 (1.0%)	1 (0.4%)	0 (0.0%)	3 (0.4%)	
Good	4 (10.5%)	1 (0.5%)	9 (4.3%)	16 (6.3%)	0 (0.0%)	30 (4.0%)	
Poor	27 (71.1%)	138 (64.2%)	150 (72.5%)	87 (34.1%)	23 (74.2%)	425 (57.0%)	
Sufficient	7 (18.4%)	76 (35.3%)	46 (22.2%)	151 (59.2%)	8 (25.8%)	288 (38.6%)	
Age Group							< 0.001
< 18	0 (0.0%)	0 (0.0%)	0 (0.0%)	209 (82.0%)	0 (0.0%)	209 (28.0%)	
18–25	10 (26.3%)	22 (10.2%)	59 (28.5%)	45 (17.6%)	5 (16.1%)	141 (18.9%)	
26–45	22 (57.9%)	119 (55.3%)	105 (50.7%)	1 (0.4%)	20 (64.5%)	267 (35.8%)	
> 45	6 (15.8%)	74 (34.4%)	43 (20.8%)	0 (0.0%)	6 (19.4%)	129 (17.3%)

**Table 3:** Knowledge/awareness of rabies and associated practices that can affect its transmission

Variables	Health worker (*N* = 38)	Farmer (*N* = 215)	Teacher (*N* = 207)	Student (*N* = 255)	Others (*N* = 31)	Total (*N* = 746)	*P* value
Total knowledge score							< 0.001
Mean (SD)	11 (2.7)	10 (2.7)	10 (3.1)	8 (3.3)	10 (3.0)	10 (3.2)	
Median (q1, q3)	12 (10.2, 13.0)	11 (9.0, 12.0)	11 (8.0, 12.0)	8 (5.0, 11.0)	11 (8.5, 12.0)	11 (7.0, 12.0)	
Range	4.0—16.0	2.0—18.0	3.0—17.0	3.0—17.0	2.0—14.0	2.0—18.0	
Standardized Knowledge score							< 0.001
Mean (SD)	57 (16.6)	51 (17.0)	51 (19.2)	38 (20.7)	50 (18.6)	47 (20.2)	
Median (q1, q3)	63 (51.6, 68.8)	56 (43.8, 62.5)	56 (37.5, 62.5)	38 (18.8, 56.2)	56 (40.6, 62.5)	56 (31.2, 62.5)	
Range	12.5—87.5	0.0—100.0	6.2—93.8	6.2—93.8	0.0—75.0	0.0—100.0	
Awareness							< 0.001
Excellent	0 (0.0%)	1 (0.5%)	2 (1.0%)	1 (0.4%)	0 (0.0%)	4 (0.5%)	
Good	2 (5.3%)	0 (0.0%)	9 (4.3%)	3 (1.2%)	0 (0.0%)	14 (1.9%)	
Poor	10 (26.3%)	76 (35.3%)	81 (39.1%)	175 (68.6%)	13 (41.9%)	355 (47.6%)	
Sufficient	26 (68.4%)	138 (64.2%)	115 (55.6%)	76 (29.8%)	18 (58.1%)	373 (50.0%)	
Total Practice score							< 0.001
Mean (SD)	4 (2.6)	5 (1.7)	4 (2.4)	5 (1.8)	4 (2.3)	4 (2.1)	
Median (q1, q3)	4 (1.0, 6.0)	5 (5.0, 6.0)	4 (1.0, 5.0)	5 (5.0, 6.0)	5 (1.0, 5.0)	5 (4.0, 6.0)	
Range	0.0—9.0	0.0—7.0	0.0—9.0	0.0—9.0	0.0—7.0	0.0—9.0	
Standardized Practice Score							< 0.001
Mean (SD)	40 (29.4)	52 (19.0)	39 (26.6)	57 (19.7)	41 (25.7)	49 (23.7)	
Median (q1, q3)	44 (11.1, 66.7)	56 (55.6, 66.7)	44 (11.1, 55.6)	56 (55.6, 66.7)	56 (11.1, 55.6)	56 (44.4, 66.7)	
Range	0.0—100.0	0.0—77.8	0.0—100.0	0.0—100.0	0.0—77.8	0.0—100.0	
Standardized practices							< 0.001
Excellent	1 (2.6%)	0 (0.0%)	3 (1.4%)	3 (1.2%)	0 (0.0%)	7 (0.9%)	
Good	4 (10.5%)	14 (6.5%)	12 (5.8%)	26 (10.2%)	1 (3.2%)	57 (7.6%)	
Poor	23 (60.5%)	50 (23.3%)	114 (55.1%)	49 (19.2%)	13 (41.9%)	249 (33.4%)	
Sufficient	10 (26.3%)	151 (70.2%)	78 (37.7%)	177 (69.4%)	17 (54.8%)	433 (58.0%)	
Age group							< 0.001
< 18	0 (0.0%)	0 (0.0%)	0 (0.0%)	209 (82.0%)	0 (0.0%)	209 (28.0%)	
18–25	10 (26.3%)	22 (10.2%)	59 (28.5%)	45 (17.6%)	5 (16.1%)	141 (18.9%)	
26–45	22 (57.9%)	119 (55.3%)	105 (50.7%)	1 (0.4%)	20 (64.5%)	267 (35.8%)	
> 45	6 (15.8%)	74 (34.4%)	43 (20.8%)	0 (0.0%)	6 (19.4%)	129 (17.3%)	

Awareness of tuberculosis among most of the survey respondents was poor (*n* = 655, 87.8%). As expected, most of the healthcare workers had better awareness of tuberculosis (Table 2, Fig. 3A) compared to students, farmers, teachers, and other professionals. TB awareness was better in participants residing in VDC 3 (18.24%) versus VDCs 1, 2, 4, and 5 (11.80%, 10.46%, 1.20%, and 13.58%, respectively).

In contrast, 50% (*n* = 373) of respondents had sufficient awareness of rabies disease (Table 3). Among the study participants, awareness of rabies was poor among students compared to other professionals (Table 3, Fig. 3B). Among respondents in the five VDCs, rabies awareness was higher in VDC 5 (69.13%) than in VDCs 1–4 (range: 33.3%—51.74%).

The mean standardized tuberculosis and rabies knowledge scores were significantly higher (*p* < 0.001) in males (37.34 ± 18.47 and 50.76 ± 18.95) compared to females (30.00 ± 17.81 and 43.85 ± 20.55) participants. However, the awareness of diseases was slightly higher in male participants than in females for tuberculosis (9.41% vs. 15.88%, *p* = 0.06, Fig. 3A) and significantly higher for rabies (male: 60.44% vs. female: 46.35%, *p* < 0.001, Fig. 3B).

As part of a zoonotic disease study, we asked participants if they were familiar with the term zoonosis. Most respondents (69.8%) did not know about zoonosis, and 65.3% of them were not sure whether TB and rabies were considered zoonotic diseases.

### Practices that affect tuberculosis and rabies transmission

The overall practice score for tuberculosis prevention was 7.76 (± 2.10) and was 4.39 (± 2.13) for rabies prevention (Tables 2 and 3). Among 746 participants, 574 (76.9%) had kept animals in their homes, with a majority disclosing the presence of cattle/buffalo (59.9%) for a source of food, income, and compost (36.1%). Most participants reported that they allow any animals in or around their homes (76.8%), but while purchasing new animals, only 3.7% quarantine them or isolate them from other animals at home. Among respondents, 73.9% mentioned that they only use household clothing as personal protective equipment (PPE) on the farm, even when handling animals. Despite many participants practicing drinking boiled milk (97.6%) at home, most participants (80.0%) also mentioned that they consume raw and unboiled milk during religious rituals. A vast majority of respondents (85.0%) answered that they do not receive regular health check-ups. Among 574 respondents who own animals, only 80 (14%) reported keeping a dog at home. Of these 80, 48.9% mentioned they vaccinated dogs against rabies, while 3/574 (0.5%) mentioned vaccinating street dogs in their VDC (1 in VDC-5 and 2 in VDC-3). Figure 3A and B indicate that those participants who had poor knowledge also scored poorly on practice and vice-versa.

### Multiple Correspondence Analysis (MCA) for relationships between categorical variables

**Fig. 2:**
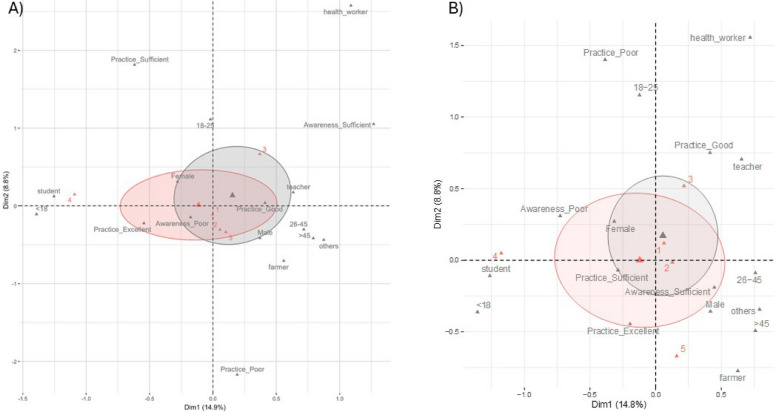
The Multiple Correspondence Analysis (MCA) plot showing demographics with (**A**) tuberculosis knowledge/Practice and (**B**) rabies knowledge/Practice. Practice is related to activities for disease prevention. Two distinct clusters are shown in red and gray colors. The red numbers indicate the sampling areas- VDCs 1–5

A multiple correspondence analysis (Fig. 2A and B, Additional file 2: Fig. 1a-1i and Fig. 2a-2i) was conducted to explore the relationships among categorical variables-Occupations, Age groups, VDCs, and Gender to visualize clustering patterns within the dataset. The MCA biplots (Fig. 2A and B) revealed two distinct clusters outlined in red and gray colors. The fact that both TB and rabies, which reveal distinct groupings across age, occupation, and rating categories, provide a nuanced understanding of how these variables interact.

### Knowledge and practices association

We explored association through correlation, regression, and mediation analysis. Pearson correlation analysis showed that tuberculosis knowledge scores have a negative correlation (−0.195, 95% CI: −0.263 to −0.125, *p* < 0.000) with practice scores to prevent TB. In contrast, rabies knowledge scores and practice scores were not correlated (0.007, −0.065 to 0.078, *p* = 0.855).

#### Regression

**Fig. 3:**
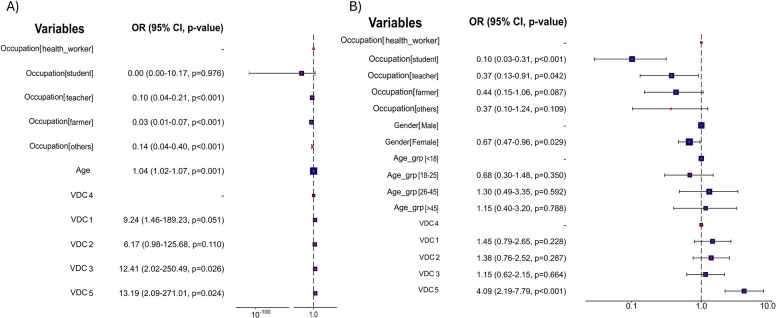
The logistic regression analysis from the multivariable logistic regression analysis of (**A**) tuberculosis awareness and (**B**) rabies awareness

**Fig. 4:**
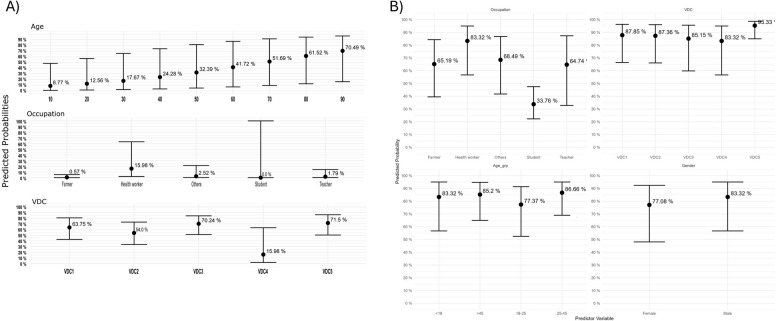
The predictive probabilities from the multinomial logistic regression analysis of (**A**) tuberculosis knowledge and (**B**) rabies knowledge

Given the importance of awareness, we recategorized the knowledge score with > 50% as the presence of TB or rabies awareness in the respondents from five VDCs and determined its association with demographic factors, as shown in Fig. 3A and B, respectively. The univariable regression results were presented in Additional File 2: Tables S1 and S2. Initial mixed-effects logistic regression model testing VDC and Occupation as random intercepts showed negligible variance (0.000), resulting in the generalized logistic regression model. With increasing age, there is a significant increase in TB awareness. TB awareness is insufficient, although it is better among healthcare professionals than in other occupations (Fig. 4A and B). In contrast, participants of all ages, VDCs, or occupations were aware of rabies, except for students.

#### Mediation and path analysis

To determine any underlying factors that may affect the knowledge of rabies and TB, we conducted the mediation and path analyses. Table 4 shows the results of the mediation analysis using 1000 bootstrap simulations and a sample of 746 participants for TB knowledge. It revealed a significant indirect effect (ACME = −0.0600, 95% CI [−0.1002, −0.02], *p* < 0.001), indicating that approximately 27% of the total effect on TB knowledge was mediated through practices. The direct effects (ADE = −0.1620, 95% CI [−0.2483, −0.07], *p* = 0.002) and total effect (TE = −0.2220, 95% CI [−0.2995, −0.14], *p* < 0.001) were also significant, confirming that factors beyond practices substantially contribute to variation in TB knowledge. The effect of practices on the TB Knowledge Score was strongly influenced by occupation, and the negative second-part effect suggests a complex relationship. Other variables did not have a mediation effect.

**Table 4:** Total, first-part, and second-part effects of respondent demographic and knowledge-score variables. Significant results were shown in orange color text

Characteristic	Total effect		First-part indirect effect		Second-part direct + indirect effects	
	β	± 95% CI**	β	± 95% CI**	β	± 95% CI**
TB Knowledge Score*	−0.13	−0.23, −0.04	−0.11	−0.21, −0.01		
Occupation	3.75	2.07, 5.43	3.27	1.54, 5.00	−6.22	−7.42, −5.02
Gender	−1.88	−4.89, 1.12	−2.427	−5.50, 0.64	−2.85	−5.10, −0.60
VDC			0.481	−0.66, 1.63	1.84	1.01, 2.67
Age			−0.14	−0.27, −0.01	0.40	0.31, 0.49
Practice Score*					−0.06	−0.11, −0.01

^*^Normalized Score

^**^CI = Confidence Interval

**Fig. 5:**
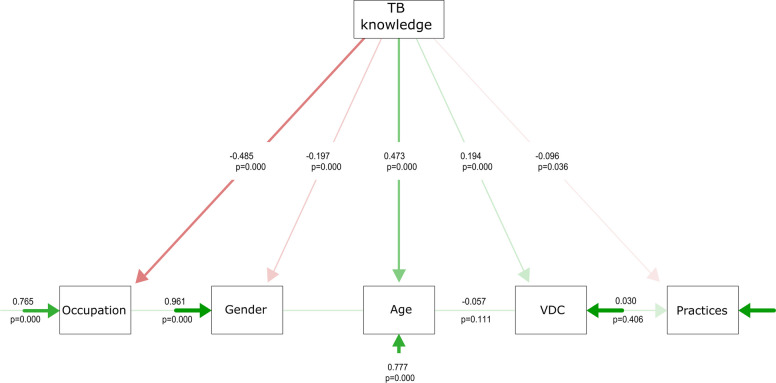
The output of the path analysis is used to determine the underlying effects of demographic factors on tuberculosis disease knowledge and associated practices

**Fig. 6:**
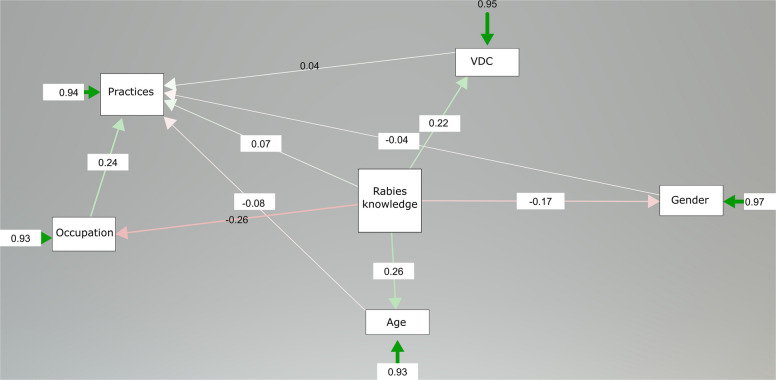
The output of the path analysis is used to determine the underlying effects of demographic factors on rabies disease and associated practices

The SEM path analysis showed that the TB knowledge (Fig. 5) model displayed a different structural pattern compared to the knowledge of rabies (Fig. 6). Occupation exerted the strongest negative influence on TB knowledge (β = −0.485, *p* < 0.001). Practices showed a weak but significant negative association with TB knowledge (β = −0.096, *p* = 0.036). In contrast, path analysis revealed that occupation was significantly associated with practices (β = 0.24), which, in turn, influenced rabies knowledge indirectly. However, the negative direct path from occupation to rabies knowledge (β = −0.08) indicates limited exposure to accurate knowledge sources despite influencing practices. Meanwhile, VDC (locality) demonstrated a positive effect (β = 0.22), suggesting community-level differences in knowledge dissemination. In both TB and Rabies knowledge, gender showed a negative association (TB: β = −0.197, *p* < 0.001; Rabies: β = −0.17), reflecting potential disparities in access to information. While age had a strong positive effect on TB (β = 0.473, *p* < 0.001) and Rabies (β = 0.26) knowledge. Age positively contributed to knowledge of rabies (β = 0.26), aligning with the assumption that older individuals may have greater cumulative exposure to disease-related information, health campaigns, or past experiences.

## Discussion

Interviews conducted within the Dhading district of Nepal provided important insights into the knowledge, attitude, and practices (KAP) surrounding tuberculosis (TB) and rabies. Despite the endemic nature of both diseases in Nepal, our findings revealed a significant gap in public awareness, particularly among youth, women, and students. General awareness of rabies was relatively higher compared to TB; however, an in-depth understanding of transmission, symptoms, treatment, and prevention of both diseases remained poor among most participants. Traditionalist values favoring males within the region could contribute to a significant knowledge gap between genders, regardless of age. These results underscore the urgent need for targeted educational and public health interventions in rural Nepal, such as in Dhading district.

Diverse facilities and participants within different VDCs may play a significant role in disease awareness. Our analysis showed that participants from VDC 5, specifically teachers, had a significantly higher knowledge score for rabies than participants from other VDCs. Later inquiry disclosed that teachers in this area received special training from a foreign education program that provided an overview of risk factors of zoonotic diseases. Training teachers could potentially be an entry point for improved awareness of TB and rabies in the Dhading district among students if the teachers pass on knowledge within the classroom. Additionally, integration of zoonosis awareness into school curricula could leverage trained teachers as knowledge multipliers [24, 30].

An incredibly low percentage (15%) of participants claimed to have regular health checkups in the study areas. This could be due to a stigma surrounding the disease in general and less access to healthcare facilities. This shows that although diseases like TB and rabies are endemic, community members do not get screened for the possibility of carrying latent TB, ultimately leading to continued endemicity. Community-based health initiatives and mobile health units or traveling healthcare professionals should be initiated to provide routine check-ups and hygiene education in remote areas [31–33]. This would help to address the persistent burden of zoonotic diseases in under-resourced regions.

Regarding the knowledge of zoonosis in the region, only 30.2% of all participants were able to define zoonosis correctly. This lack of understanding of the possibility of disease transmission from animals to people is a troubling statistic, given that most of the population of the Dhading district is in close contact with animals daily. Even many healthcare workers (63.2%) could not correctly define zoonosis, calling into question the level of training dedicated to infectious diseases. It also indicated there is a dire need for the latest or refresher training about zoonoses and infectious diseases to combat emerging and re-emerging diseases. Initiation and continuation of cross-sector collaboration between human and animal health services would help to improve such disease surveillance and response to zoonotic threats [15, 18, 30, 34].

Over half of the participants claim to live near cattle or buffalo (59.9%), which can harbor bovine tuberculosis, a strain of TB capable of zoonosis. Rural towns that rely on these animals for milk or meat are at significant risk for zoonotic disease transmission, given that they are unaware of the risk of disease and the precautions necessary to avoid transmission into the community. When analyzing the farmers' practices in handling animal products such as milk, surveys showed that they use limited hygiene practices. In addition to direct contact with animals, it has been reported that milk products are one of the potential sources of TB spread in rural areas. Findings of other studies also aligned with poorly adopted prevention practices and lower TB awareness in farmers compared to highly reported outbreaks such as rabies and bird flu [6, 35–37]. Proper educational training of people, especially farmers, on food and food product hygiene and disease prevention could reduce the transmission of TB.

Rabies also continues to plague the Dhading district, with an average of 10 animal rabies outbreaks annually [14, 38]. The large stray dog population, which is disproportionate to the human population in Nepal, likely contributes to the frequency of rabies outbreaks, as vaccinating stray animals would prove difficult and costly in an under-resourced and rural area [3, 8, 10]. However, it may be more cost-efficient in the long term to mass vaccinate the stray dog population [43]. One Health strategies have been effective in neighboring countries such as Bhutan, Bangladesh, and Sri Lanka. From 2006 to 2026, Bhutan only had seventeen reported cases of rabies. Bangladesh targeted rural communities and then later integrated into the rest of the nation using a One Health multi-sectoral approach. They reduced rabies deaths from 1500 in 2012 to 200 in 2015. Additionally, Sri Lanka was able to decrease rabies deaths to fewer than 50 in 2012 through the use of mass vaccination in dogs [44].

We found that the demographics who know the least about rabies (women and youth) have the highest chances of encountering any animal. Although not many human rabies cases have been reported in Dhading district, animal outbreaks pose a significant threat to the vulnerable demographics in the region. All the youth in our population were students under the age of 18 years. Community education programs should move beyond basic awareness to emphasize the importance of timely vaccination and proper bite management. We recommend incorporating rabies education into the school curriculum, which can be disseminated by teachers. Teachers and healthcare workers can serve as key advocates for rabies prevention, leveraging their trusted roles to raise awareness and promote behavior change [23–25]. A study conducted in a rural, mountainous area of central Sri Lanka demonstrated the effectiveness of an educational campaign. A lecture and rabies awareness pamphlet were distributed in class. Knowledge scores increased with both the lecture and the pamphlet rather than with the pamphlet alone [45]. A child-focused meta-analysis also found that short, developmentally appropriate interventions delivered in schools yield large improvements in rabies safety knowledge and practices [39]. Programs such as UNICEF-supported school interventions also underscore the impact of teacher-led rabies education in Sub‑Saharan Africa (Côte d’Ivoire: Community-led rabies education). Future health education and awareness campaigns should therefore focus strategically on these groups.

Mediation and pathway analysis have been increasingly used in KAP studies. This enables us to further determine the effect of each variable on the knowledge assessed. The rabies knowledge path analysis in our study revealed a complex interplay between practices, occupation, age, gender, and VDC. When comparing rabies and TB models, occupation emerged as a key determinant, but through different mechanisms. For TB, occupation had a strong, direct negative influence on knowledge acquisition. Healthcare workers often show higher TB awareness compared to agricultural or informal sector workers, but gaps persist due to stigma and misconceptions. A one-health approach should be employed to combat tuberculosis in both humans and animals, given the evidence of both bovine and human transmission in both directions.

Interestingly, the negative second-part mediation effect suggests that some occupational groups may demonstrate practices that paradoxically reduce TB knowledge or reflect conflicting information sources. Other sociodemographic variables did not exert mediation effects, suggesting their role in TB knowledge acquisition may be more direct. Thus, while general community campaigns remain valuable, tailoring interventions to occupational contexts could yield stronger outcomes. In addition, behavioral practices partially shape knowledge levels, highlighting the bidirectional influence between knowledge and practices in TB prevention and control. For rabies, however, occupation influenced practices directly and indirectly shaped knowledge through behavioral exposure.

Gender disparities were evident in TB knowledge but less prominent in rabies knowledge, suggesting disease-specific sociocultural dynamics. The negative association of gender with rabies knowledge underscores a key divergence from TB results, where gender differences were not a strong mediator. This may reflect cultural patterns in which women, especially in rural and agricultural contexts, have fewer opportunities to receive rabies-specific education despite playing central roles in animal care and family health decisions. These findings mirror studies from South Asia and Africa, which reported lower rabies knowledge among women and low-income workers despite high exposure risk [40–44].

Both models emphasized the importance of age and community-level factors (VDC) as enablers of knowledge of TB and rabies. Taken together, the two models suggest that while TB knowledge is strongly mediated through practices and occupational context, rabies knowledge is more directly shaped by demographic (age, gender) and community (VDC) characteristics. This divergence highlights the need for disease-specific educational strategies: workplace-based interventions may be more effective for TB, whereas rabies control requires community-wide and gender-sensitive approaches that prioritize local health systems, outreach in rural villages, and intersectoral “One Health” strategies to effectively bridge the gap between knowledge and practices in TB and rabies control programs.

These findings reveal that all demographics within the Dhading district could benefit from TB and rabies education programs. Implementing mandatory health education programs with emphasis on zoonotic diseases in schools could improve awareness and the health of students and other demographics in the Dhading district. Teaching children in schools about the warning signs of rabies in animals could be a feasible intervention that can save lives. In rural areas, such as in the Dhading district, general health check-ups could be a vital intervention, especially for farmers at the highest risk of catching and transmitting zoonotic diseases. A traveling healthcare professional could provide general health maintenance checks and education in proper hygiene around animals. However, the stigma around illness, as well as the quick turnaround of healthcare workers, hinders countrywide health initiatives from taking hold in Nepal. Educating the population on the importance of consulting a healthcare provider could be a critical step in reducing the disease burden in the region; however, due to the region's rural and marginalized nature, access to care needs to be addressed.

The first rabies immunization in Nepal was launched in 1983, and several free anti-rabies vaccine programs have been launched with national goals to eliminate rabies by 2030 [3]. This study was conducted among people recovering from the massive earthquake that had destroyed many areas, including the study sites in Dhading. During that period, eight districts, including Dhading, had > 10 rabies outbreaks [3]. Currently, the political map of the district has changed, and the availability of resources has improved, including technology. However, Nepal still has reported a rabies outbreak that claimed three deaths in 2025. A recent report showed the TB incidence rate in Nepal was 229 (126–355) per 100,000 population [45]. There is also a persistent gap in TB knowledge, detection, and treatment coverage in various regions of Nepal [3, 15, 34, 35]. Hence, our study results remain valid, emphasizing that the regular implementation of KAP studies in rural and less accessible healthcare areas improves the health literacy of the general population for disease prevention.

Additionally, this study was conducted before Nepal’s decentralization reforms. Decentralization has shifted certain responsibilities to local governments, and VDCs are being reorganized and reassigned to municipalities [46, 47]. The shift of responsibilities to seven provincial governments and 753 local governments has granted greater autonomy over health service planning and delivery. Technological shifts are also changing the information flow since the promulgation of Nepal’s 2015 Constitution and subsequent decentralization. However, the core issues identified, such as knowledge gaps, attitudes toward health practices, and behavioral patterns, continue to influence program implementation [47, 48]. This continues to pose significant hurdles for local governments as they implement the National Health Policy and formulate tailored municipal health strategies. Local authorities have reported improvements in infrastructure, outreach, and data reporting; however, they continue to face persistent challenges, including coordination across tiers, delayed finance flows, staffing shortages, and inadequate monitoring systems [46–49]. Our findings thus serve as an essential baseline to inform tailored community education campaigns, capacity-building programs, and strengthened knowledge-to-practice translation, all of which are crucial to support the transformative potential of Nepal’s health decentralization for better community health outcomes.

Our sample size was relatively large; however, the proportion of healthcare workers and participants classified in the “Others” group was significantly smaller than that of students, teachers, and farmers. Healthcare workers play a crucial role in preventing tuberculosis (TB), both human and animal TB, as well as rabies. In the context of bovine TB (*Mycobacterium bovis*), healthcare professionals must recognize the zoonotic risk among individuals with occupational exposure to cattle or dairy products. Studies report latent TB infection rates as high as 10% among dairy workers, emphasizing the need for integrated screening programs and PPE use in livestock environments [50]. Similarly, other healthcare workers, especially community pharmacists, are uniquely positioned to interact with patients more frequently than physicians and are well-situated to offer rabies vaccination services and follow-up care protocols. This is particularly true for the rural areas of low- and middle-income countries. By combining enhanced vaccine access, protocol-driven PEP administration, and patient education, pharmacists and other health workers serve as key community assets in translating rabies awareness into timely and effective preventive action [51]. Regular training and baseline screening for healthcare workers, combined with prompt evaluation after exposure, are essential for early detection and prevention. Awareness of large-scale assessment of zoonotic diseases in different healthcare settings is further warranted in Nepal.

This study was conducted in certain participant groups that can play a pivotal role in reducing the rabies and TB burden in Nepal. However, the survey was limited to rural areas, and not all occupations were included. Additionally, we have not assessed the educational level of adult participants, which may affect the results and is outside the scope of this study. Technology development has changed the educational environment, even in rural areas. We presented the baseline study and recommended collecting and evaluating the influence of the educational level and other occupations on rabies and TB-related behavior modification and their prevention in the future. Because participants were either illiterate or had a lower literacy level due to age, surveys had to be conducted verbally, which could introduce bias through inconsistent wording between translators. Nevertheless, such bias has been addressed through study design and various statistical analyses.

Zoonotic diseases are complex and shaped by human behavior, animal host and its behavior, and changes in their environment, and require ethical approaches that extend beyond single discipline perspectives [52]. Our study was conducted when formal ethical approval from the national ethical review board was not mandated for veterinary and/or zoonotic research. It is essential to acknowledge that ethical standards for research involving animals and zoonotic diseases in Nepal have undergone substantial evolution since then [53]. Under current frameworks, similar studies would typically require formal review and approval by both nationally recognized medical and veterinary ethics committees to safeguard human and animal welfare and maintain research integrity. The updated Nepal Health Research Council guidelines further enhance protections for minors and vulnerable populations, as seen in this study, by requiring guardian consent and child assent with explicit justification, tailored risk–benefit assessments, clear communication, and proactive measures to prevent coercion or exploitation [53]. Future research should adhere to contemporary ethical principles in veterinary and public health research practices in Nepal, aligning with international norms. Simultaneously, we recommend developing zoono-ethics [52] that integrates principles from human and veterinary research ethics, focusing on the risk–benefit balance, human-animal welfare, community engagement, cultural sensitivity, and precaution under uncertainty, while adapting to local socio-ecological contexts. These improved guidelines and integrated approach foster scientifically based and socially responsible research, address the critical public health needs, while upholding human rights, animal welfare, public trust, and strengthening national ethical review systems.

## Conclusions

Zoonotic diseases continue to disproportionately affect developing countries like Nepal due to a critical gap in knowledge, awareness, and practices related to tuberculosis and rabies among the public. Rural communities, such as those in the Dhading district, are especially vulnerable to these diseases due to the increased likelihood of exposure to both domesticated and wild animals found around farms. Women, youth, and students were identified as high-risk groups with the lowest awareness levels. Gender disparity in health education and limited access to care put individuals at a greater risk of contracting and spreading these diseases, with little opportunity to seek medical attention. An improved understanding of zoonoses and greater access to healthcare could be a vital first step in reducing the burden of TB and rabies in the Dhading district and other parts of Nepal. The low understanding of zoonosis, even among healthcare workers, highlights a critical need for improved education and training. Overall, our findings highlight the necessity of workplace- and occupation-specific health education. Embedding disease awareness programs within professional settings may help bridge knowledge gaps, correct misconceptions, and improve practices in high-risk groups.

## Supplementary Information

Supplementary Material 1.

Supplementary Material 2.

## Data Availability

The raw datasets used and/or analyzed in this study are not publicly available due to the individual privacy of the participants. However, de-identified data can be made available from the corresponding author on reasonable request.

## Abbreviations

AIDS: Acquired immunodeficiency syndrome
BCG: Bacille Calmette-Guérin
HICAST: Himalayan College of Agricultural Sciences and Technology
HIV: Human immunodeficiency
HRIG: Human rabies immunoglobulin
KAP: Knowledge, attitudes and practices
KS: Knowledge scores
MDR: Multidrug-resistant
PPE: Personal protective equipment
TB: Tuberculosis
UICOMP: University of Illinois College of Medicine
VDC: Village Development Committees
