# Supplementary material for: Awareness and practices survey of two ancient zoonotic diseases in Dhading District of Nepal
Source: BMC Public Health. 2026 Mar 7;26:1216. doi: 10.1186/s12889-026-26757-y (PMC13081520; doi:10.1186/s12889-026-26757-y)
Supplement: Supplementary file 1 — Supplementary Material 1. [file 12889_2026_26757_MOESM1_ESM.pdf]

Additional file 1: Survey Questionnaire

Name: Age:

Gender:

Address: Profession:

Regarding Farm Management

1.Is there any farm animals/pets present in your household?

i) Yes ii) No

2. How many animals of which species do you owe?.................................

3. How animals are reared in your farm?

i)Stall fed ii) Free range iii) Both

4. What are the sources of water for animals in your farm?

i)Tap water ii) Well water iii) Pond water iv) River water

5. What do you do while working in the animal farm?

i)Wear farm clothes ii) Wear gloves and boots

iii)Wear regular boots iv) others_______

6. How do you clean animal houses?

i) Remove dung ii) Clean floor with water iii) Use disinfectants

7.What are the things you do after working in farm?

i)Clean hand and leg with regular water

ii)Clean hand and leg with soap water

iii)Use disinfectants

iv)Don't do anything

8. How do you dispose farm waste?

i)Throw in pit ii) Direct disposal in farmland iii) Composting

ii) Bio gas v) Others

9. Are there any water resources near a farm or grazing area?

i)Well ii) Pond iii) River iv) Stream water

10. If yes, what is the water resource?.......................

11. How far is the water resource located?..

12. Do you vaccinate your animal?

i)Yes ii) No

13. Against which disease have you vaccinated your animal?................................

14. Do you do health check up of animal prior to buying?

i)Yes ii) No

15. Do you practice quarantine of newly bought animals?

i)Yes ii) No

16. If yes, how many days are you in quarantine?

i) <2 days ii) 2-5days iii) 5-10days iv) >10days

17. Who takes care of animals most of the time?

i) Both

ii) Male member of family

ii) Female member of family

iii) Depends on accessibility of time

Environment and Food Hygiene

1. What do you do before milking animals?

i)Clean udder ii) Clean shed iii) Both(i) and (ii) iv) None

2. How do you consume milk?

i) Raw ii) Boiled iii) Sometime raw usually boiled

3. How often do you consume raw milk?

i) Never ii) During religious ritual only iii) Occasionally iv) Often

4. How often do you consume meat?

i) Not at all ii) During festivals only iii) Twice a week iv) Once a month

5. From where do you buy meat?

i) Local butcher ii) Meat shop iii) Animal slaughtered in village iv) all of them

6. How do you consume meat?

i)By grilling ii) By pressure cooking iii) By boiling iv) Raw

7. Do you consume condensed meat/milk products?

i)Yes ii) No

8. Do you know you can become infected with zoonotic disease if you consume raw meat and milk products?

i)Yes ii) No

9. What are the diseases that can be transmitted from consuming raw meat/milk?......

Zoonoses

1.What is zoonoses?

i)Disease transmitted among animals

ii)Disease transmitted from animal to human

iii)Disease transmitted from human to animals

iv)Both (i) and (ii)

v)Unknown

2. Do you know name of any zoonotic diseases?........................

3. Can you tell how zoonotic diseases are transferred?

i)By direct contact with animal and secretions

ii)Consuming raw animal products

iii)Through urine and faeces of animal

iv)All of above

v)No idea

4. Who are at more risk of zoonotic disease?..

5. Do you let your kids to play with pet or farm animals?

i)Yes ii) No

DISEASE KNOWLEDGE

Rabies

1.Have you heard about rabies?

i)Yes ii) No

2. Do you know what rabies is?................................

3. Who are susceptible to rabies?

i)Cattle/Buffalo ii) Dog iii) Human iv) Wild animals v) All of the above

4.How is it transmitted?

i)Dog bite ii) Saliva of infected animal

iii)Jackal bite iv) Consumption of milk and meat from infected animal

v)All vi) None

5. What are the symptoms shown by rabies animals?..............................

6.What are the symptoms shown by rabies infected human?............................

7. Have you vaccinated your pet dog?

i)Yes ii) No

8. If yes, when did you last vaccinated?...........................

9. Are the street dogs in your community vaccinated against rabies?

i)Yes ii) No

10. What do you think you should do in cases of dog bite?

i)Wash with soap and water ii) Wash with water

iii)Use disinfectants iv) Go to nearest health center

11. Is rabies curable or not?

i)Yes ii) No

Bovine Tuberculosis

1. Have you heard about bovine tuberculosis?

i)Yes ii) No

2.Have you heard about tuberculosis in human?

i) Yes ii) No

3. Who are susceptible to tuberculosis?

i) Cattle ii) Goat iii) Human iv) Buffalo v) Pig vi) All

4. What are the ways of transmission of tuberculosis?

i) Through direct contact

ii) Contaminated secretions

iii) Consuming raw milk

iv) All

5. What are the symptoms seen in case of tuberculosis?

i)Frequent coughing ii) Anorexia iii) Become lean and thin

iv)Blood in sputum v) Fever vi) Weight loss

vii) Black skin color viii) All

6. Is your animal tested for bovine tuberculosis?

i)Yes ii) No

7. What system is affected by tuberculosis in human?

i)Respiratory ii) Circulatory iii) Urinary iv) All

8. Is anyone in your family suffering from tuberculosis?

i)Yes ii) No

9. If yes, is s/he getting treated?

i)Yes ii) No
