# Supplementary material for: Awareness and practices survey of two ancient zoonotic diseases in Dhading District of Nepal
Source: BMC Public Health. 2026 Mar 7;26:1216. doi: 10.1186/s12889-026-26757-y (PMC13081520; doi:10.1186/s12889-026-26757-y)
Supplement: Supplementary file 2 — Supplementary Material 2. [file 12889_2026_26757_MOESM2_ESM.pdf]

Figure S1a: Screeplot for tuberculosis

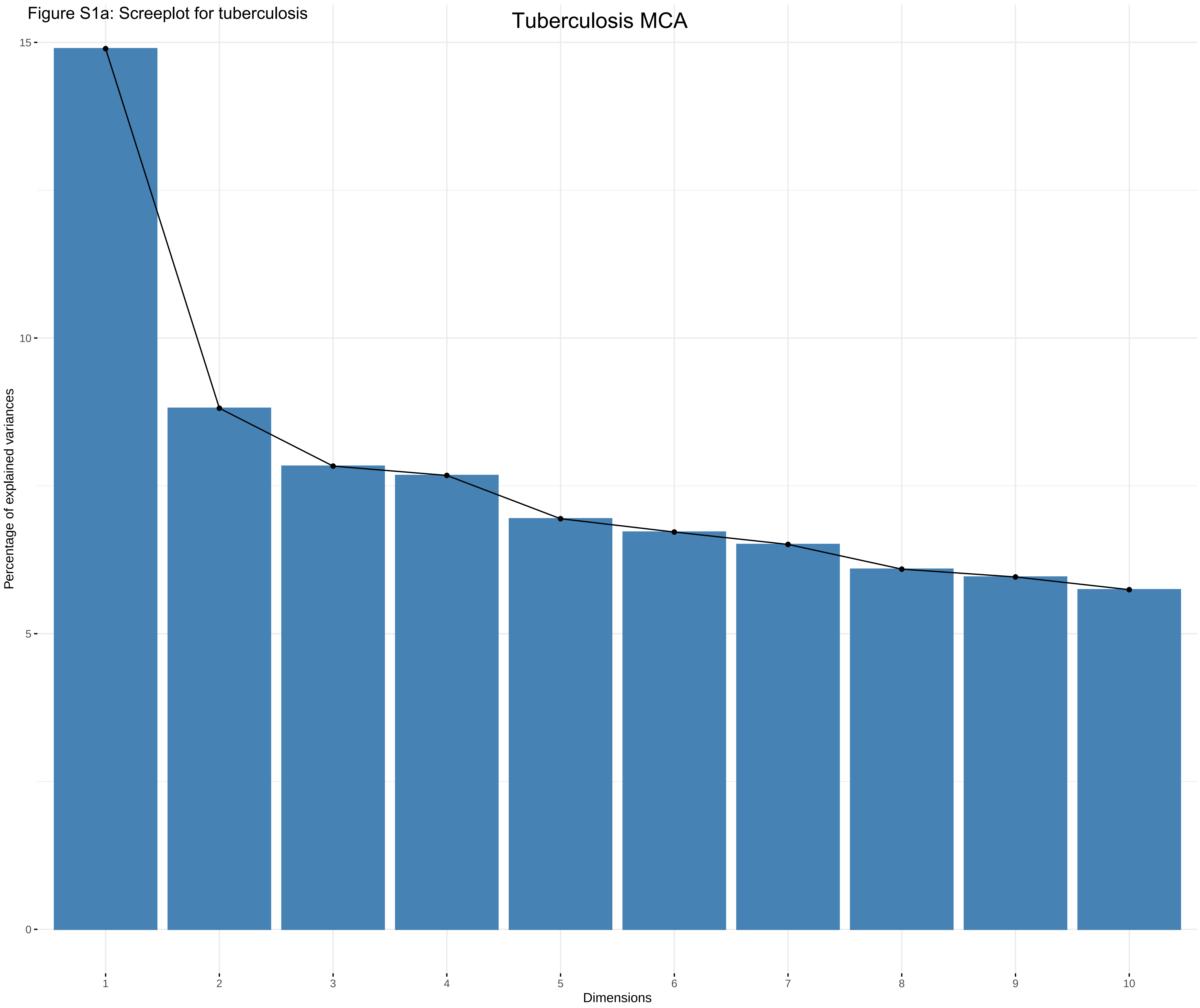

Figure S1b: Biplot for tuberculosis

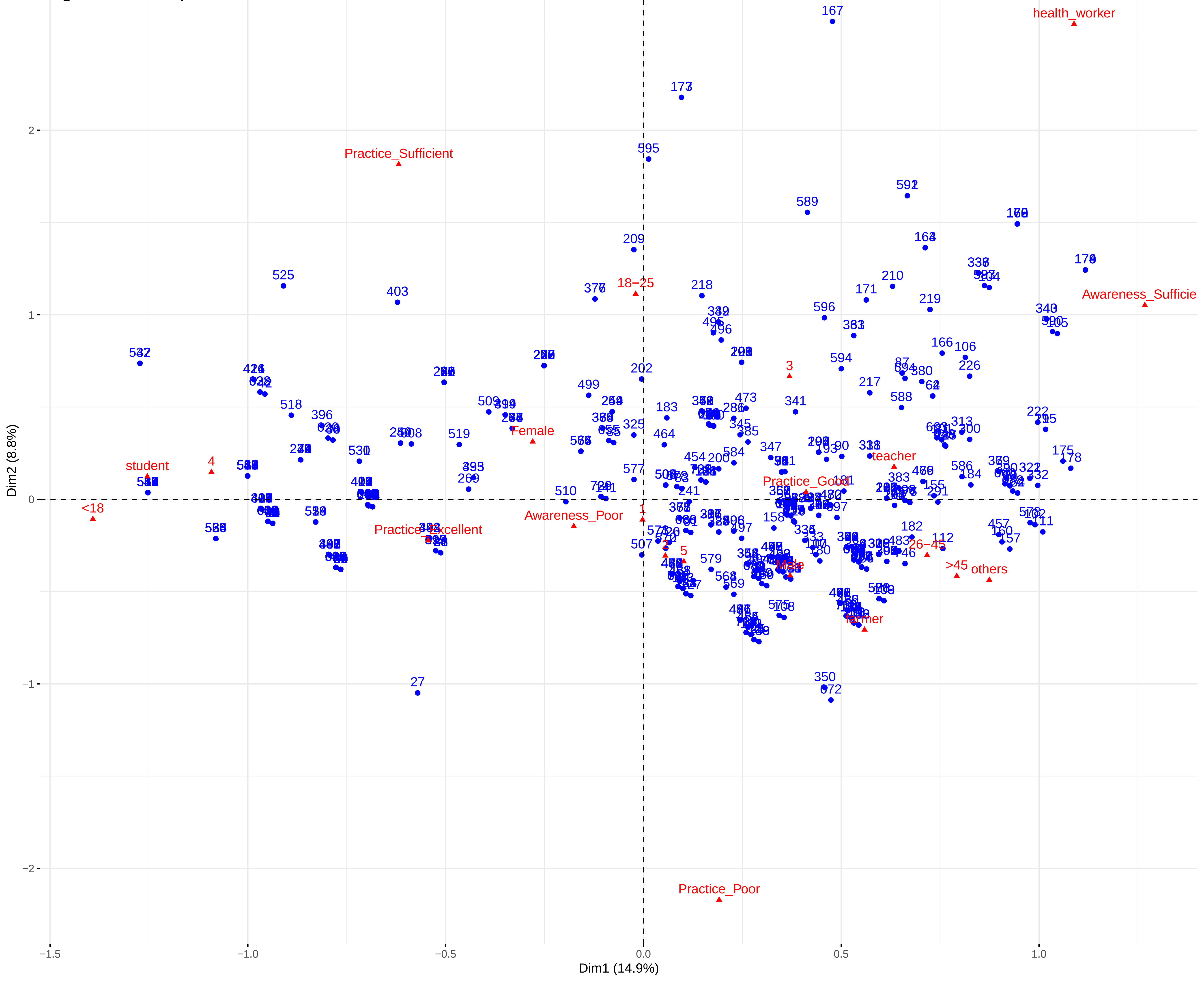

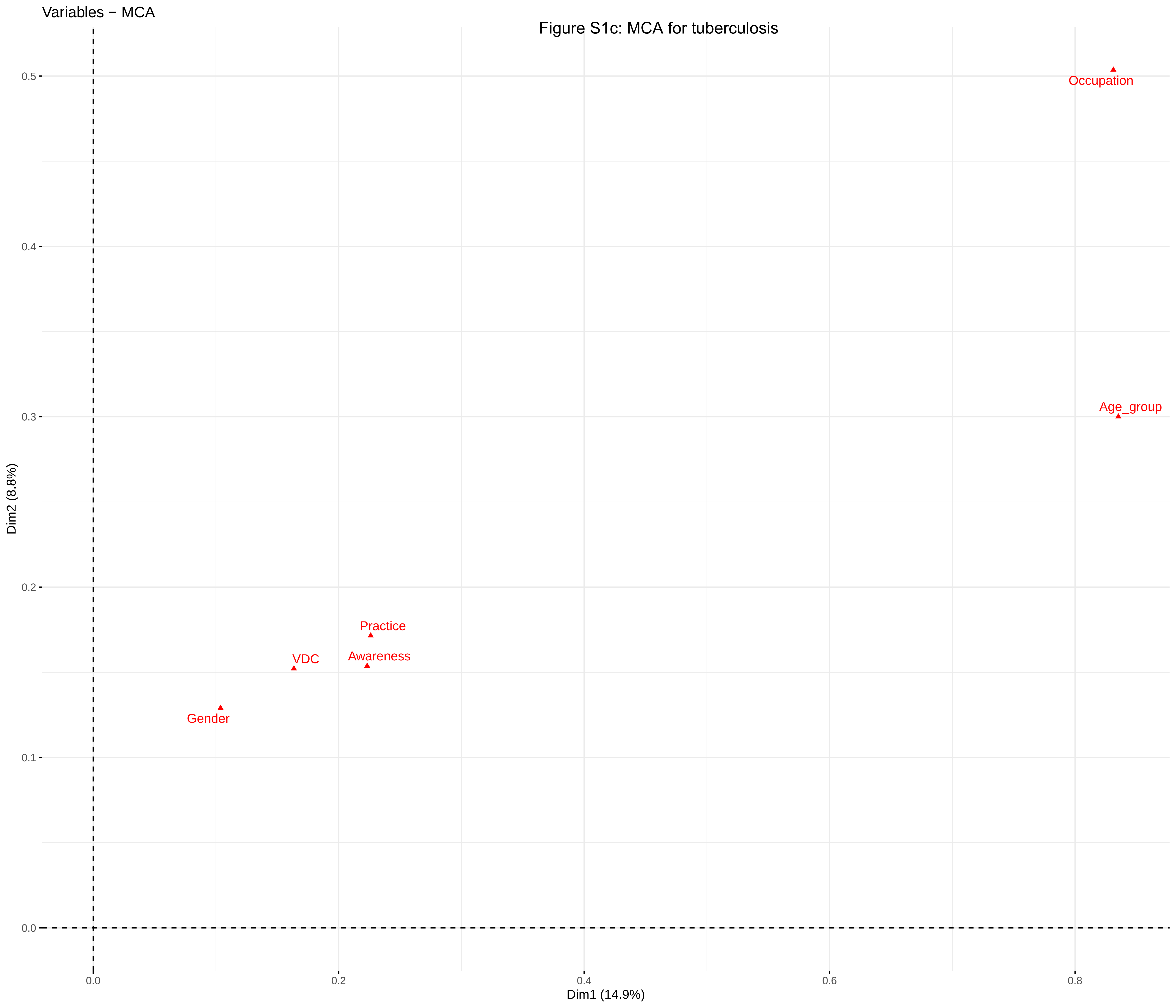
Variables − MCA
Figure S1c: MCA for tuberculosis
Occupation
Age_group
Practice
VDC
Awareness
Gender
Dim2 (8.8%)
Dim1 (14.9%)
0.0
0.1
0.2
0.3
0.4
0.5
0.0
0.2
0.4
0.6
0.8

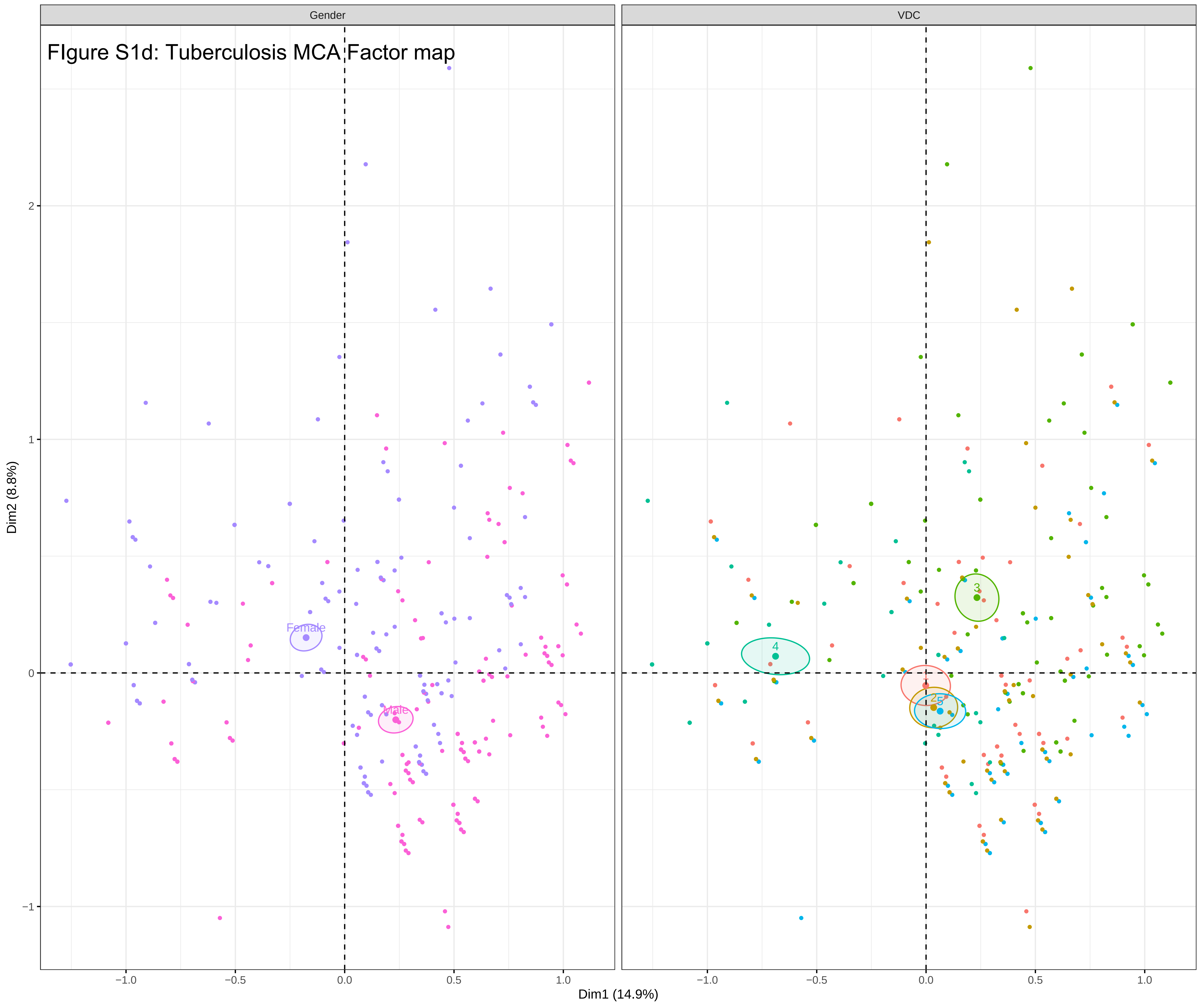
Gender
VDC
FIgure S1d: Tuberculosis MCA Factor map
Female
Male
1
2
3
4
5
Dim2 (8.8%)
Dim1 (14.9%)
2
1
0
−1
−1.0
−0.5
0.0
0.5
1.0

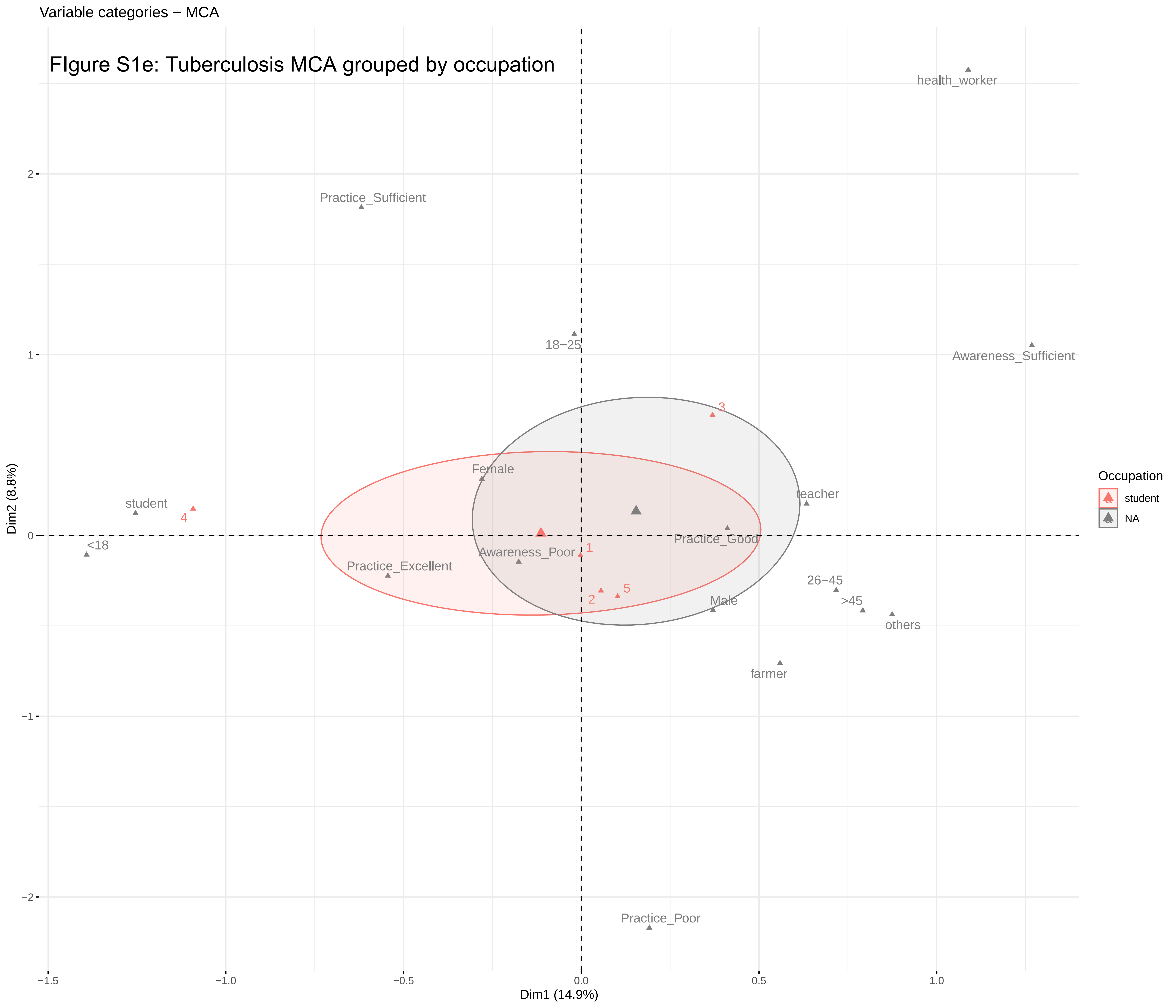
Variable categories − MCA
FIgure S1e: Tuberculosis MCA grouped by occupation
health_worker
Practice_Sufficient
18−25
Awareness_Sufficient
3
Female
teacher
student
4
<18
Practice_Good
Awareness_Poor
1
Practice_Excellent
26−45
5
2
Male
>45
others
farmer
Practice_Poor
Dim1 (14.9%)
Dim2 (8.8%)
Occupation
student
NA
−1.5
−1.0
−0.5
0.0
0.5
1.0
2
1
0
−1
−2

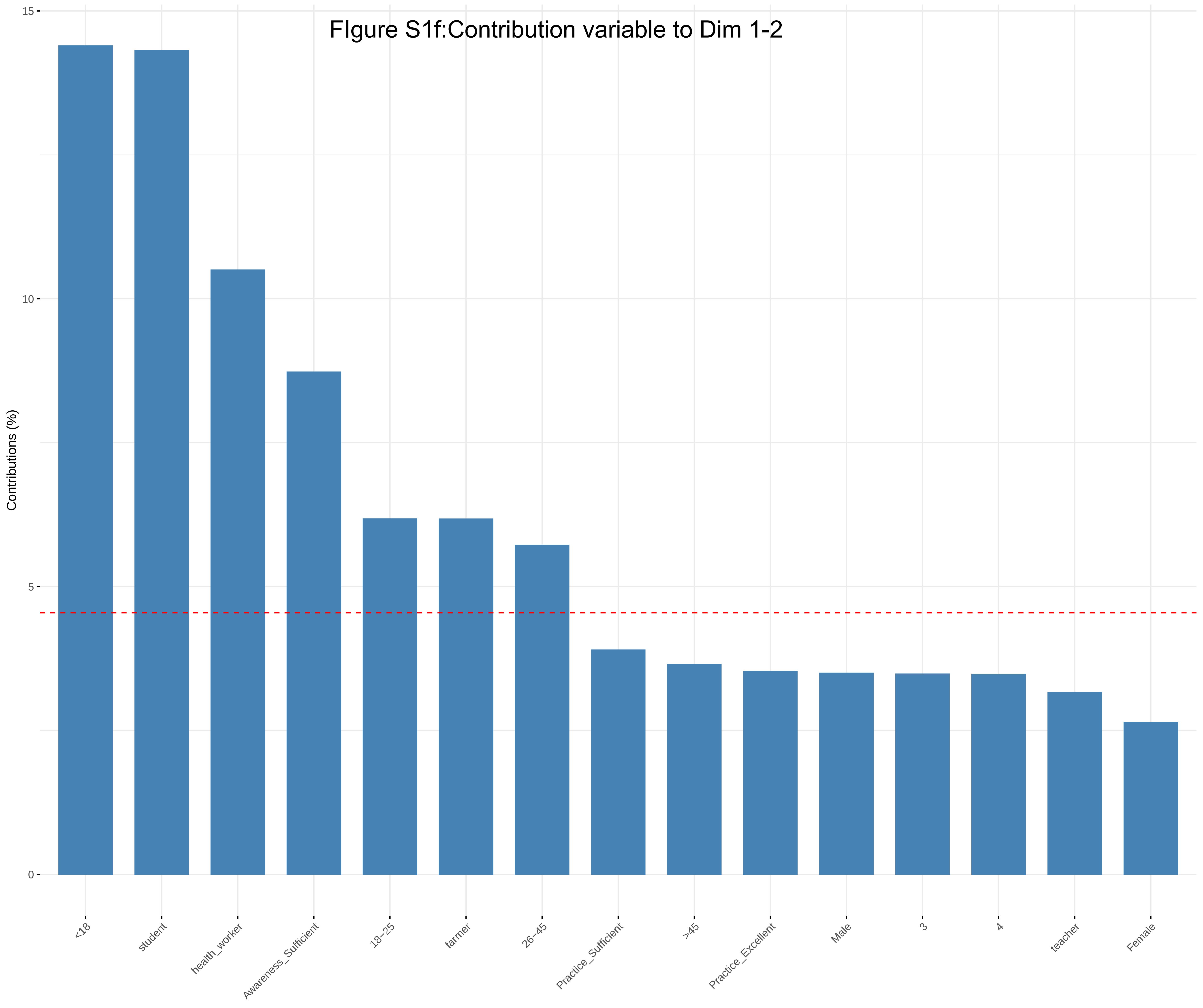
FIgure S1f:Contribution variable to Dim 1-2
Contributions (%)
0
5
10
15
<18
student
health_worker
Awareness_Sufficient
18–25
farmer
26–45
Practice_Sufficient
>45
Practice_Excellent
Male
3
4
teacher
Female

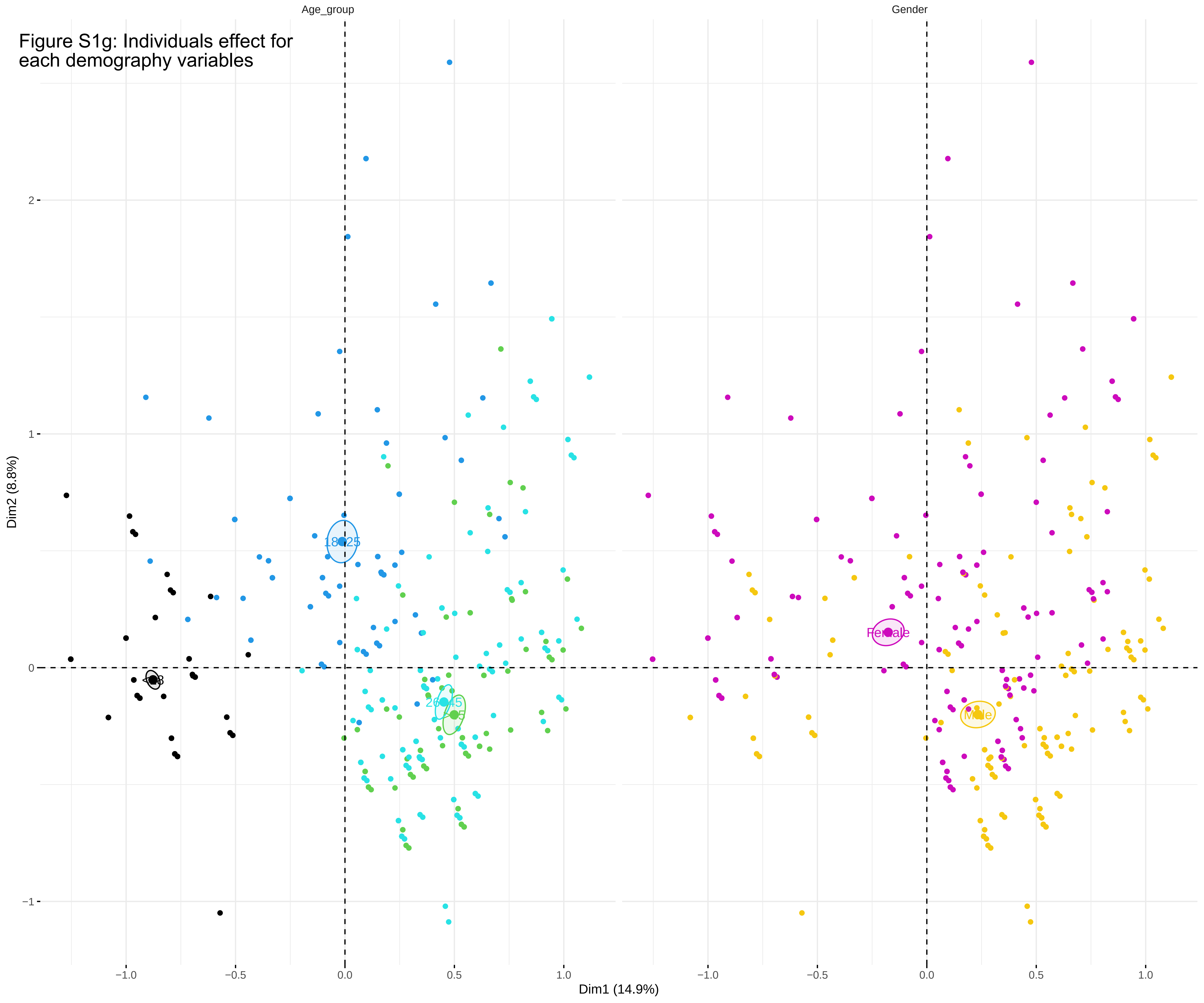

Figure S1g: Individuals effect for
each demography variables
Age_group
Gender
18-25
26-45
Female
Male
Dim1 (14.9%)
Dim2 (8.8%)

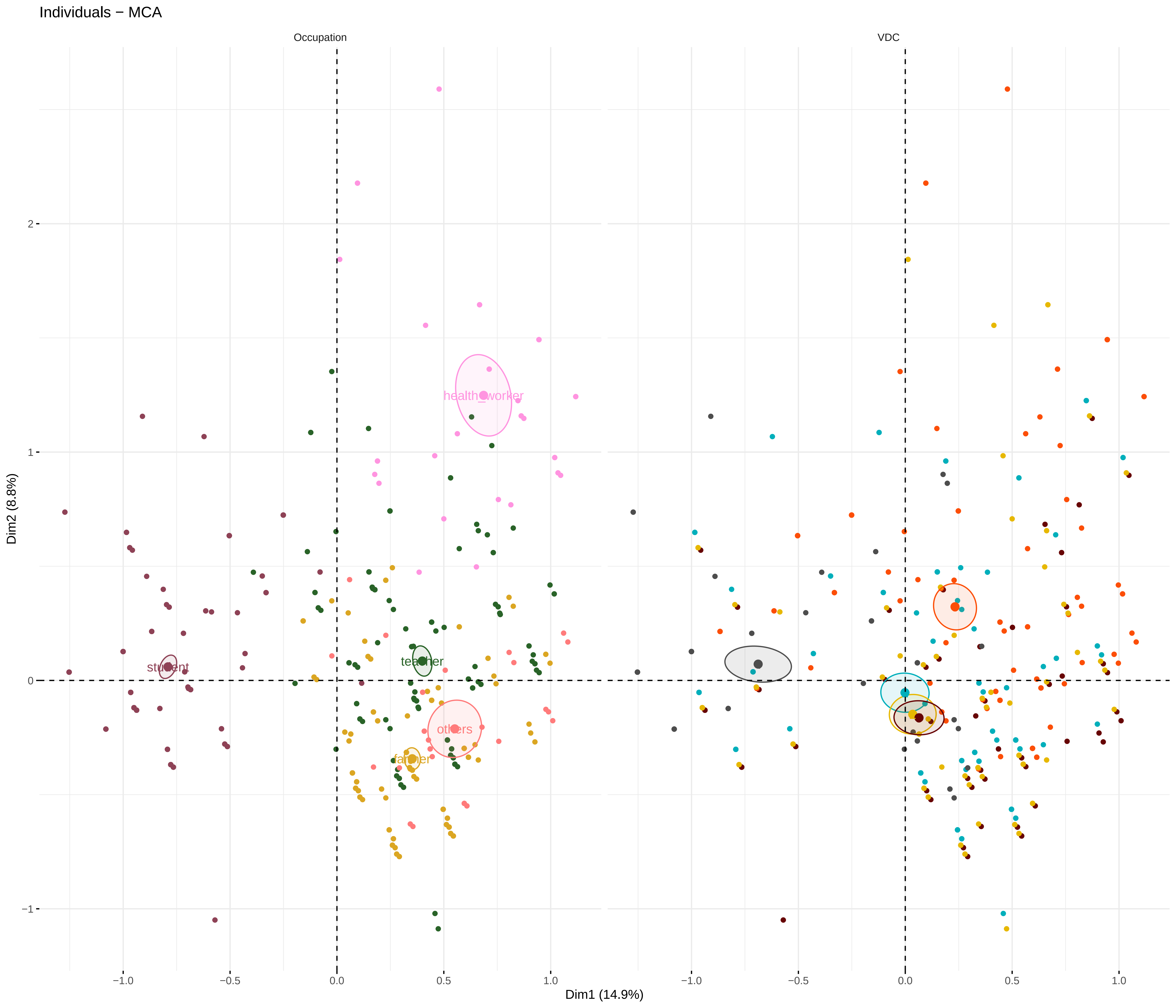
Individuals − MCA
Occupation
VDC
health_worker
teacher
student
others
farmer
Dim1 (14.9%)
Dim2 (8.8%)
−1.0
−0.5
0.0
0.5
1.0
2
1
0
−1

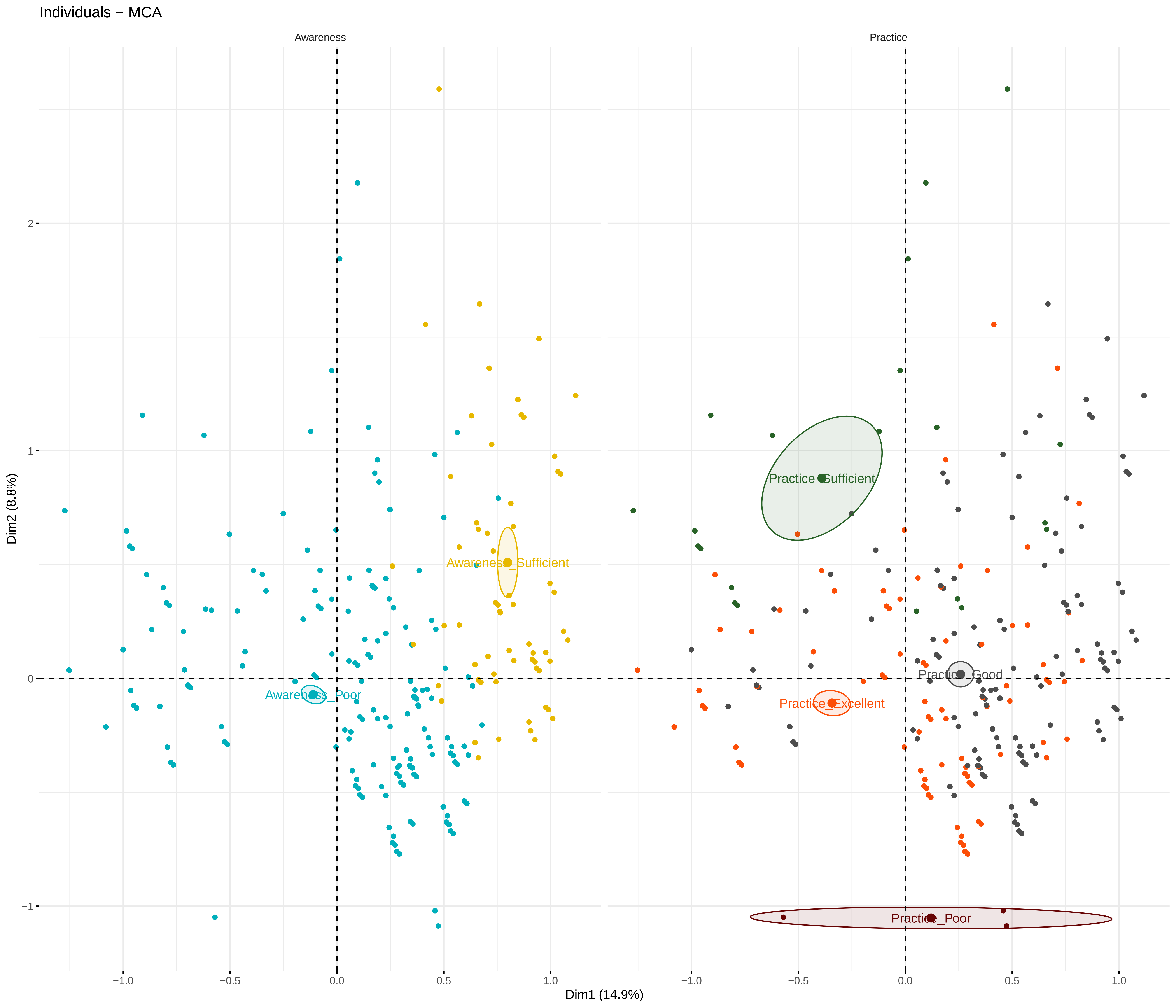

Individuals − MCA
Awareness
Practice
Awareness_Sufficient
Awareness_Poor
Practice_Sufficient
Practice_Good
Practice_Excellent
Practice_Poor
Dim1 (14.9%)
Dim2 (8.8%)
−1.0
−0.5
0.0
0.5
1.0
2
1
0
−1

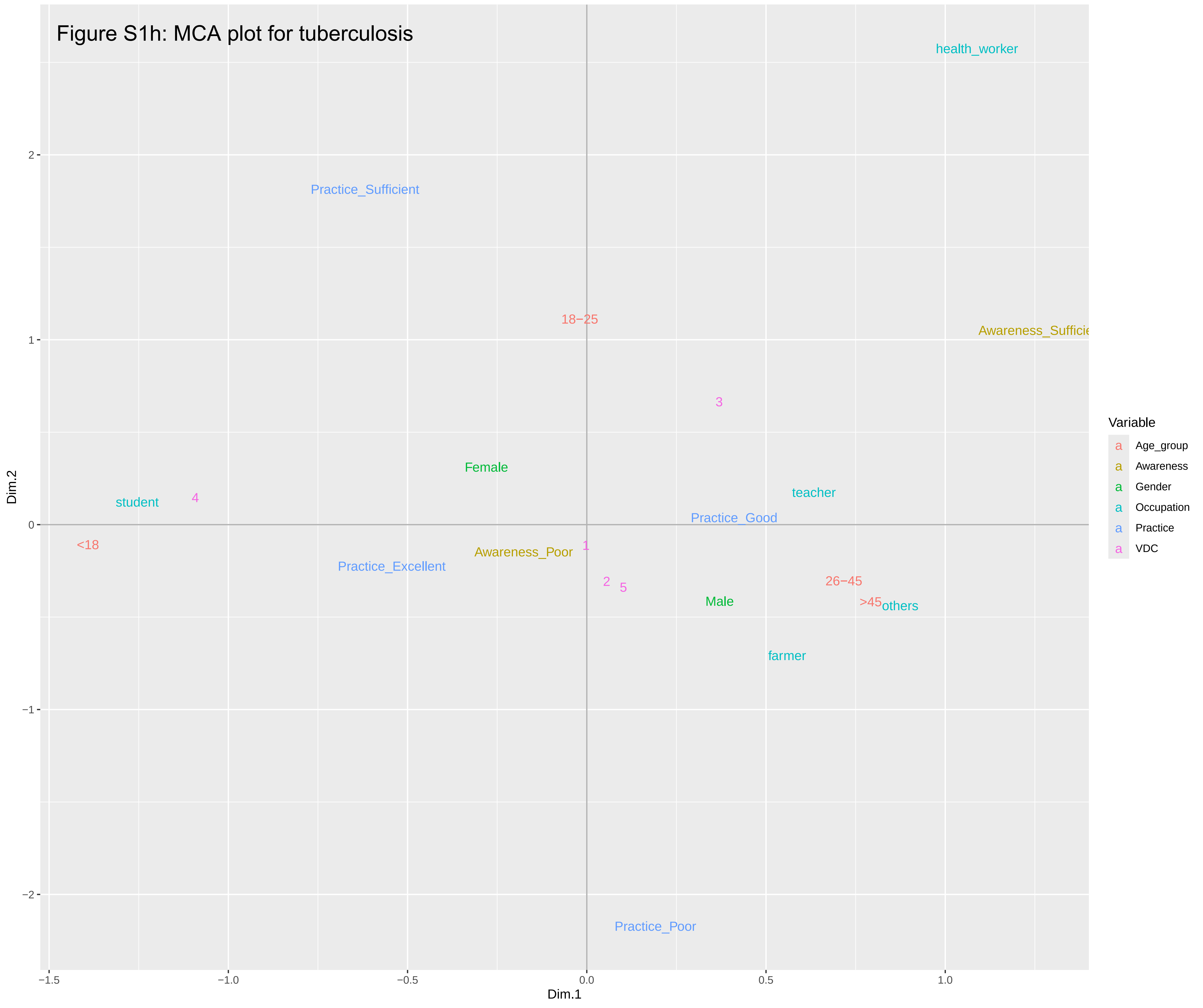
Figure S1h: MCA plot for tuberculosis
health_worker
Practice_Sufficient
18−25
Awareness_Sufficie
3
Female
student
4
teacher
Practice_Good
<18
Awareness_Poor
1
Practice_Excellent
2
5
26−45
Male
>45
others
farmer
Practice_Poor
Dim.1
Dim.2
Variable
Age_group
Awareness
Gender
Occupation
Practice
VDC

Figure S1i: Density plots for tuberculosis MCA

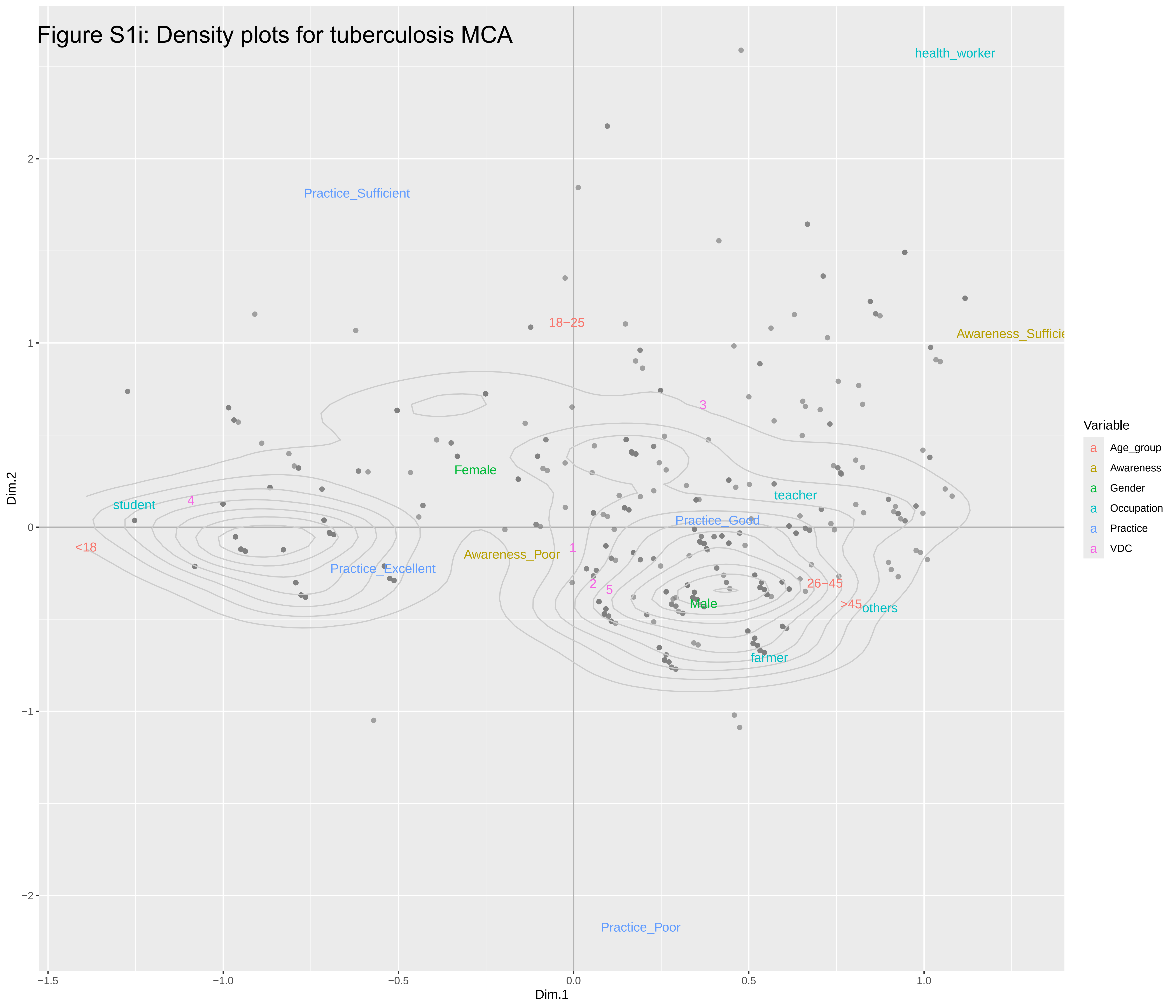

Figure S2a: Scree plot for Rabies MCA

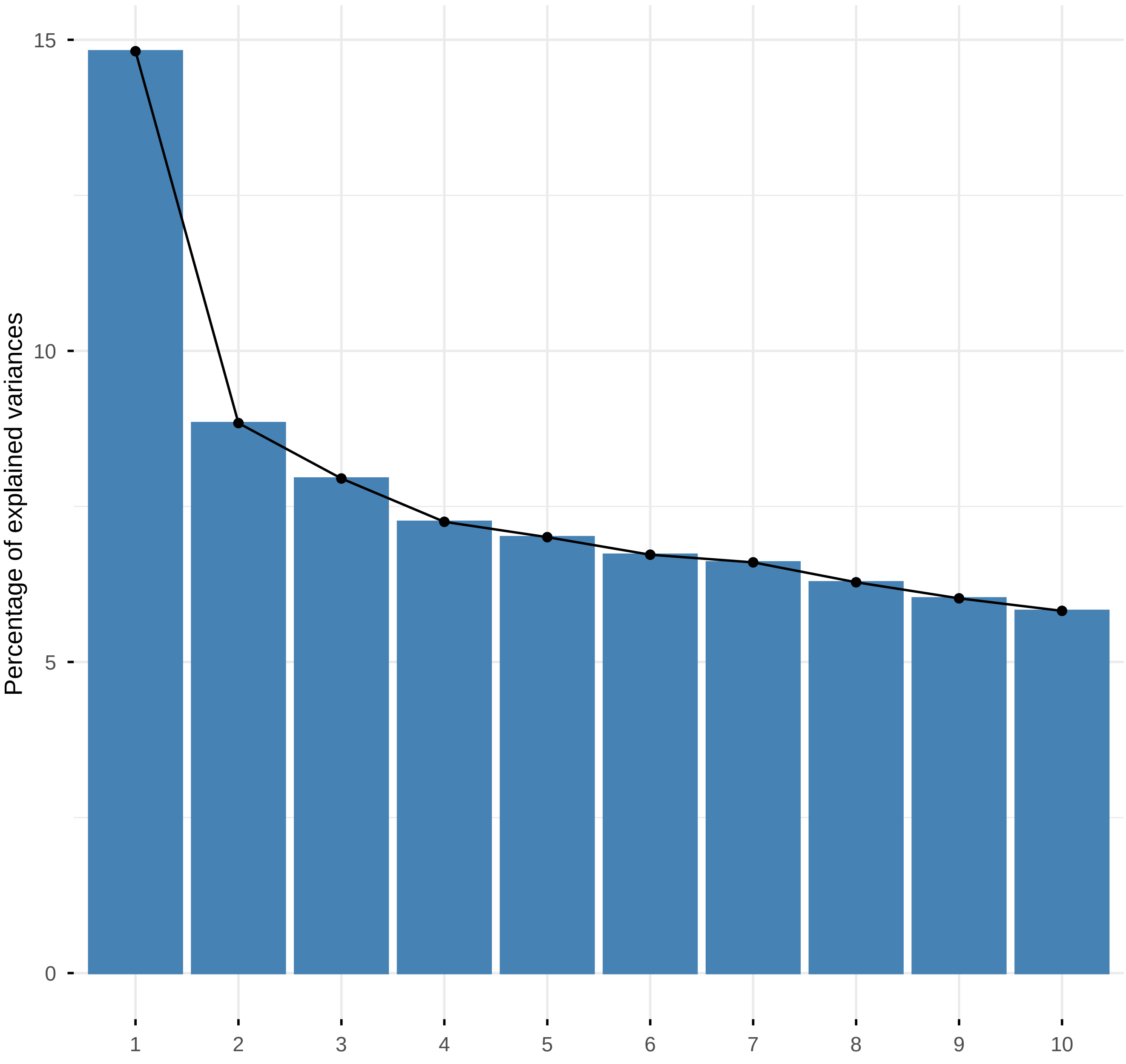

Dimensions

Figure S2b: Rabies MCA Biplot

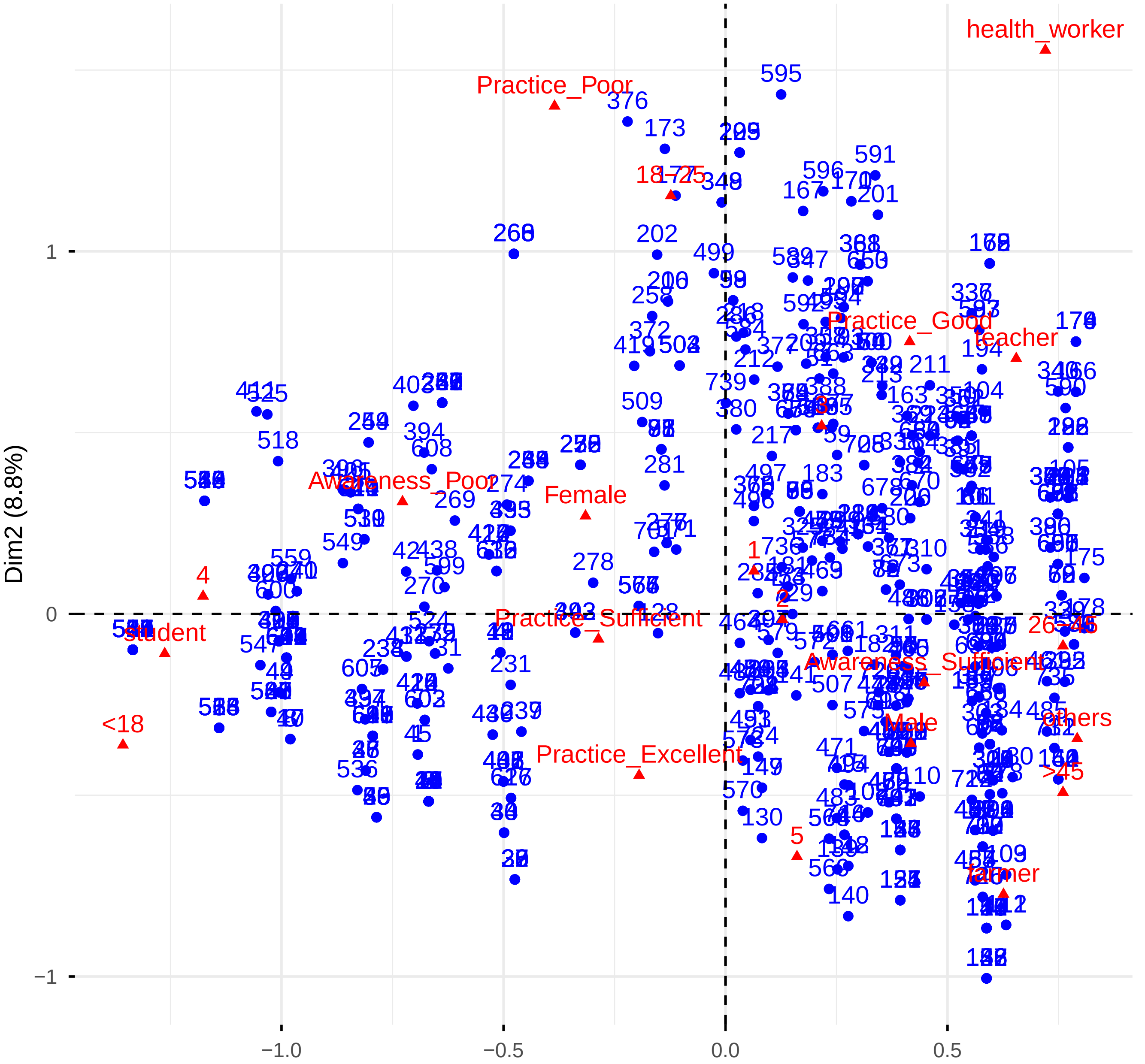

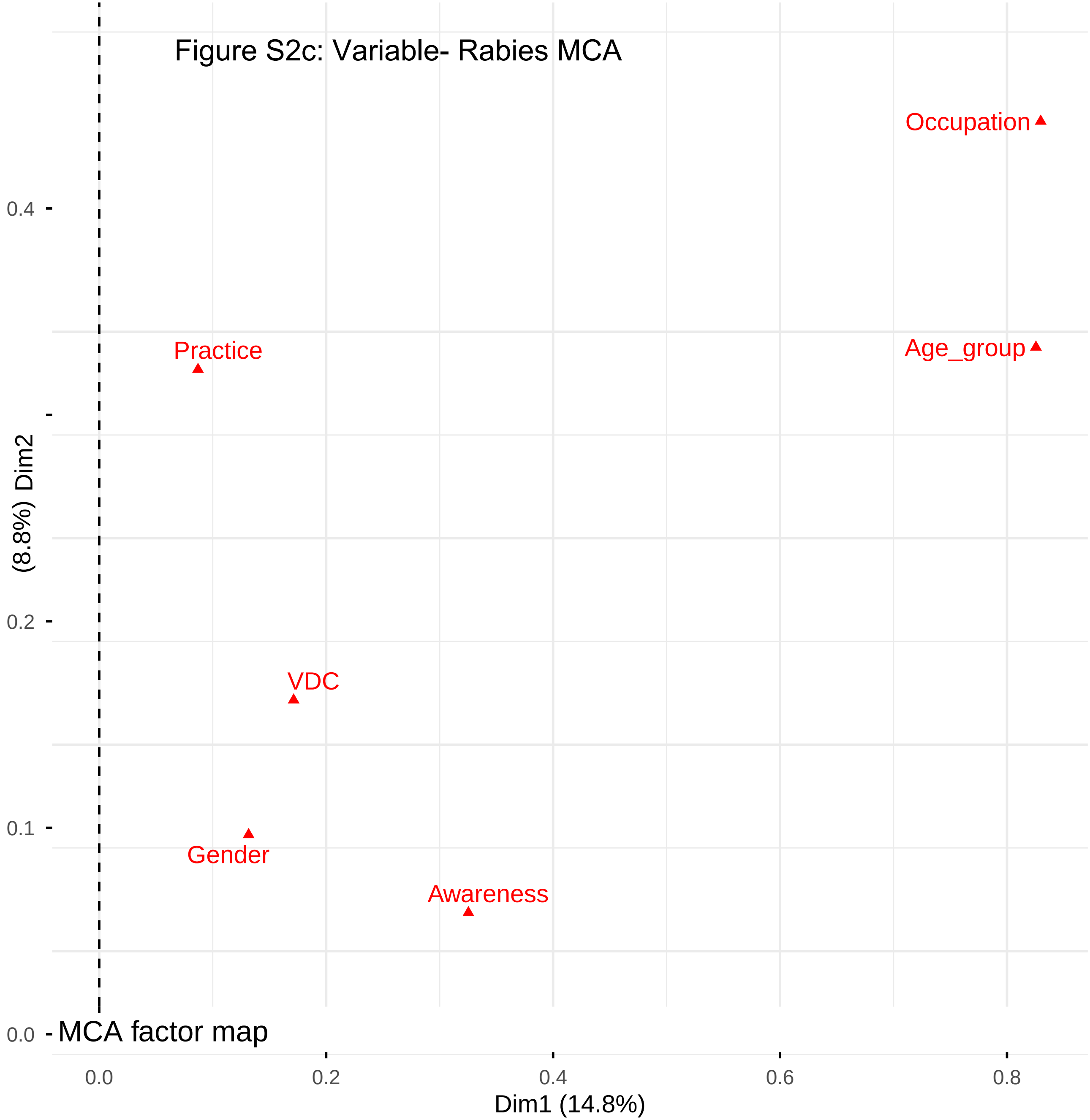
Figure S2c: Variable- Rabies MCA
Occupation
Age_group
Practice
VDC
Gender
Awareness
MCA factor map
0.4
0.2
0.1
0.0
(8.8%) Dim2
0.0
0.2
0.4
0.6
0.8
Dim1 (14.8%)

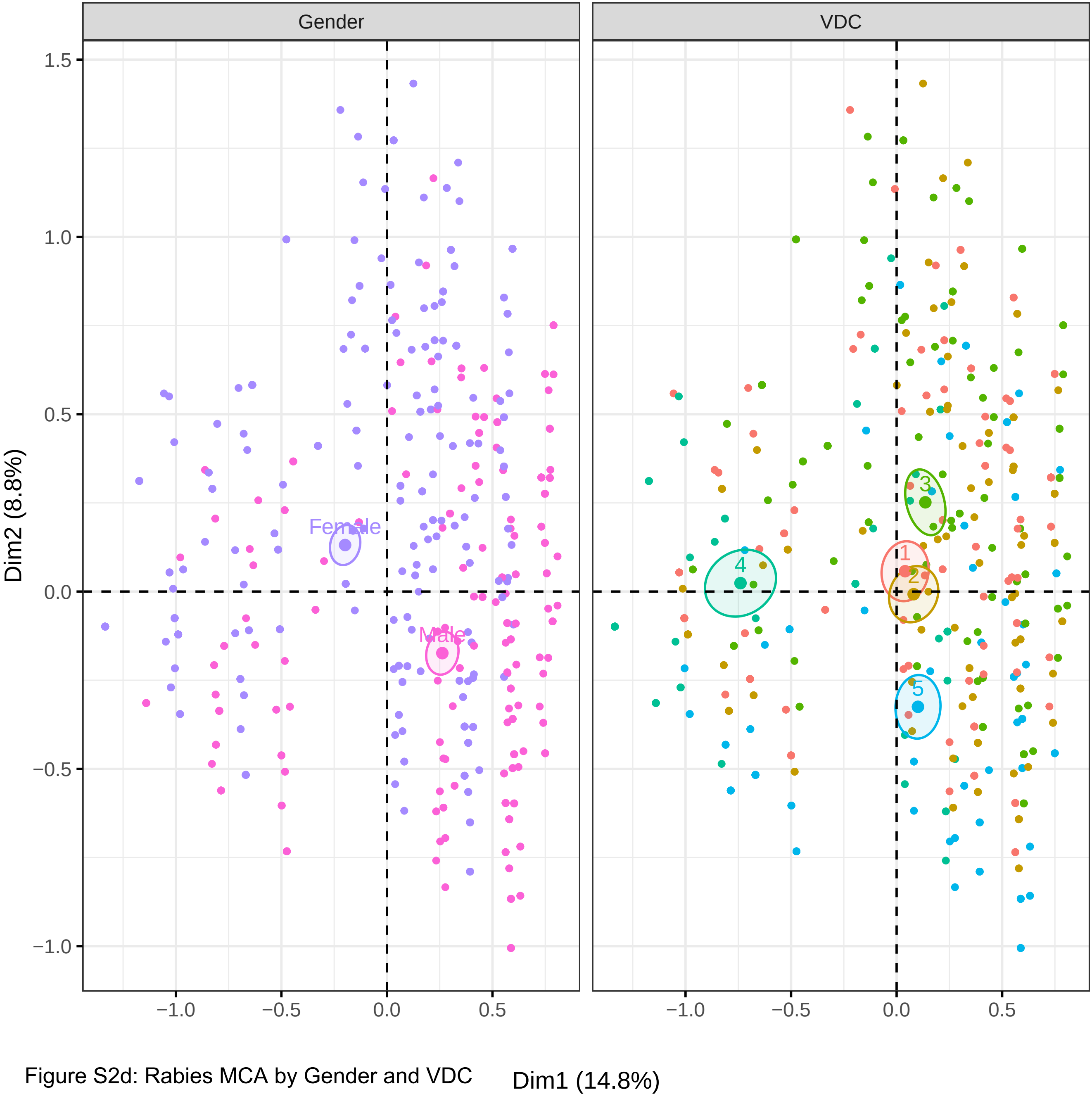

Figure S2d: Rabies MCA by Gender and VDC

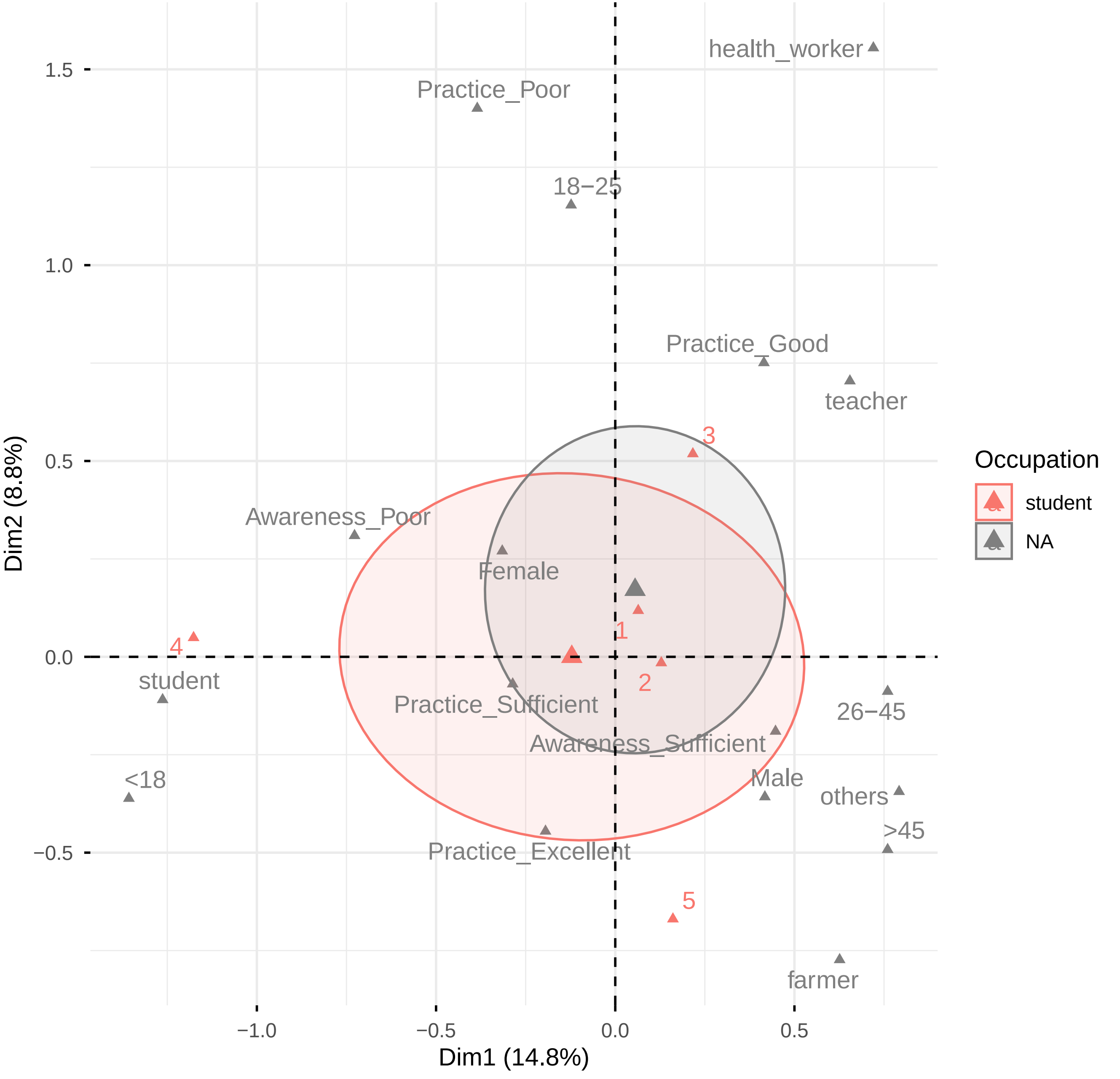

Figure S2e: Variable categories − Rabies MCA

Figure S2f: MCA Contribution of variables to Dim−1−2

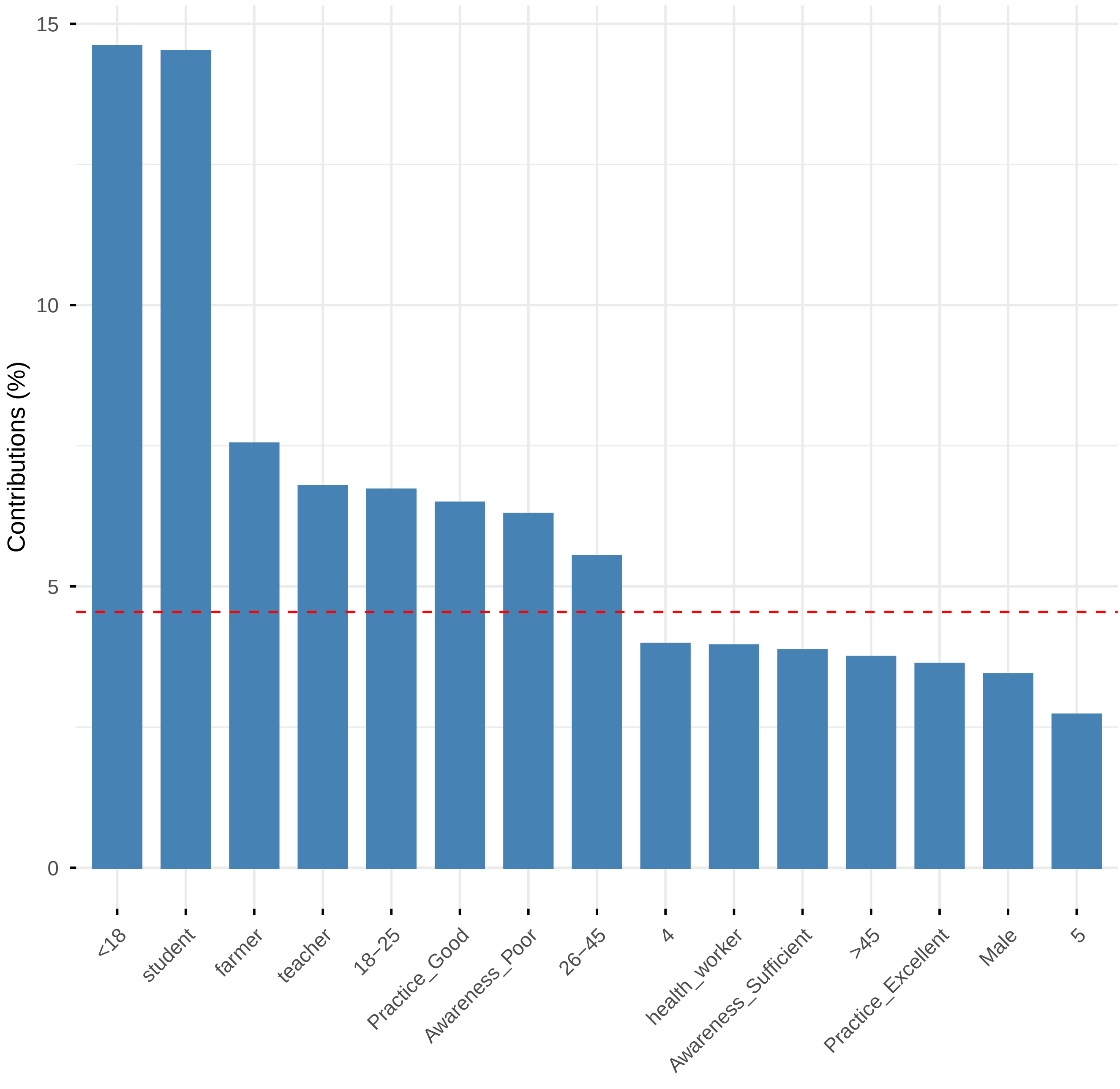

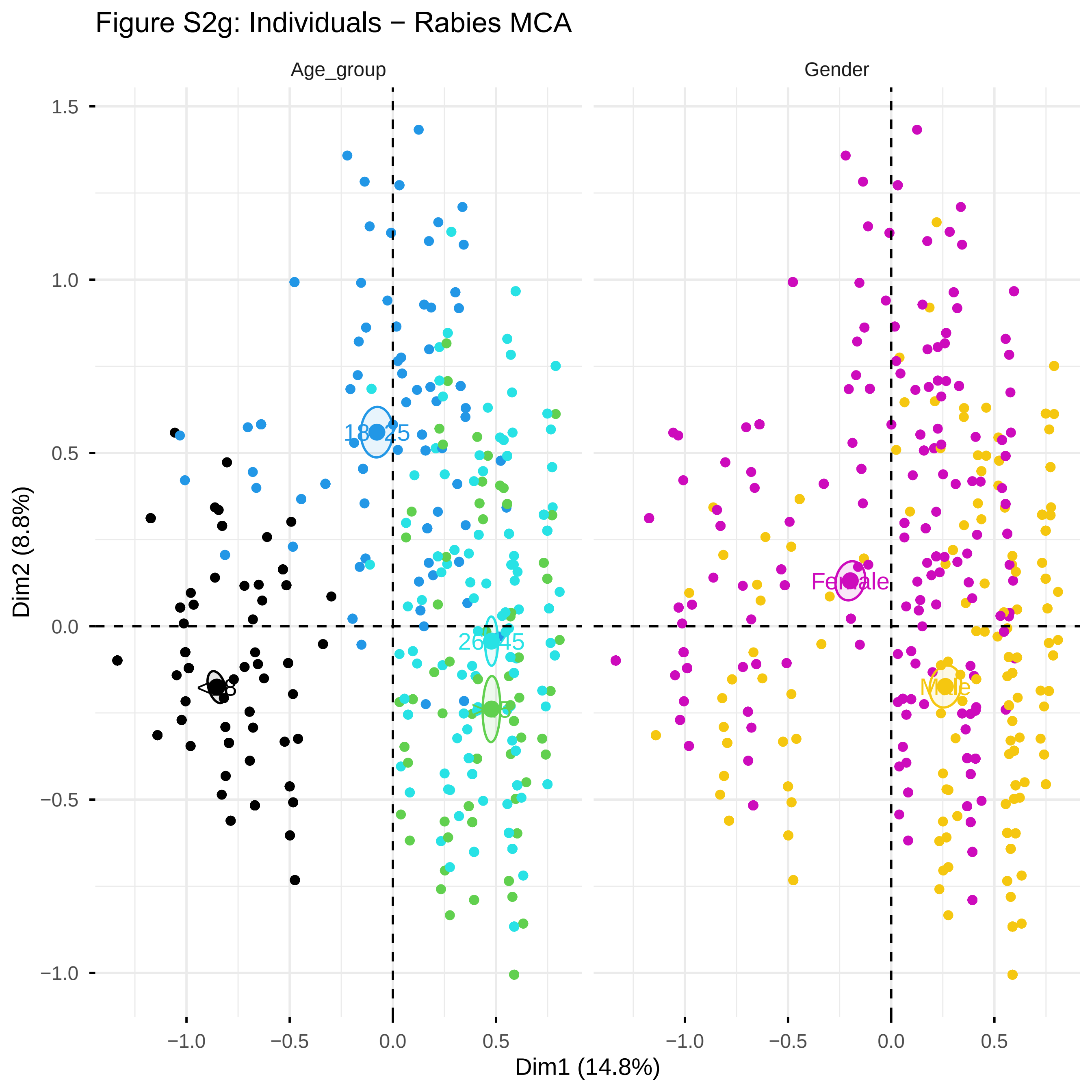

Figure S2g: Individuals − Rabies MCA
Age_group
Gender
Dim2 (8.8%)
Dim1 (14.8%)
1.5
1.0
0.5
0.0
−0.5
−1.0
−1.0
−0.5
0.0
0.5
18-25
26-45
Female
Male

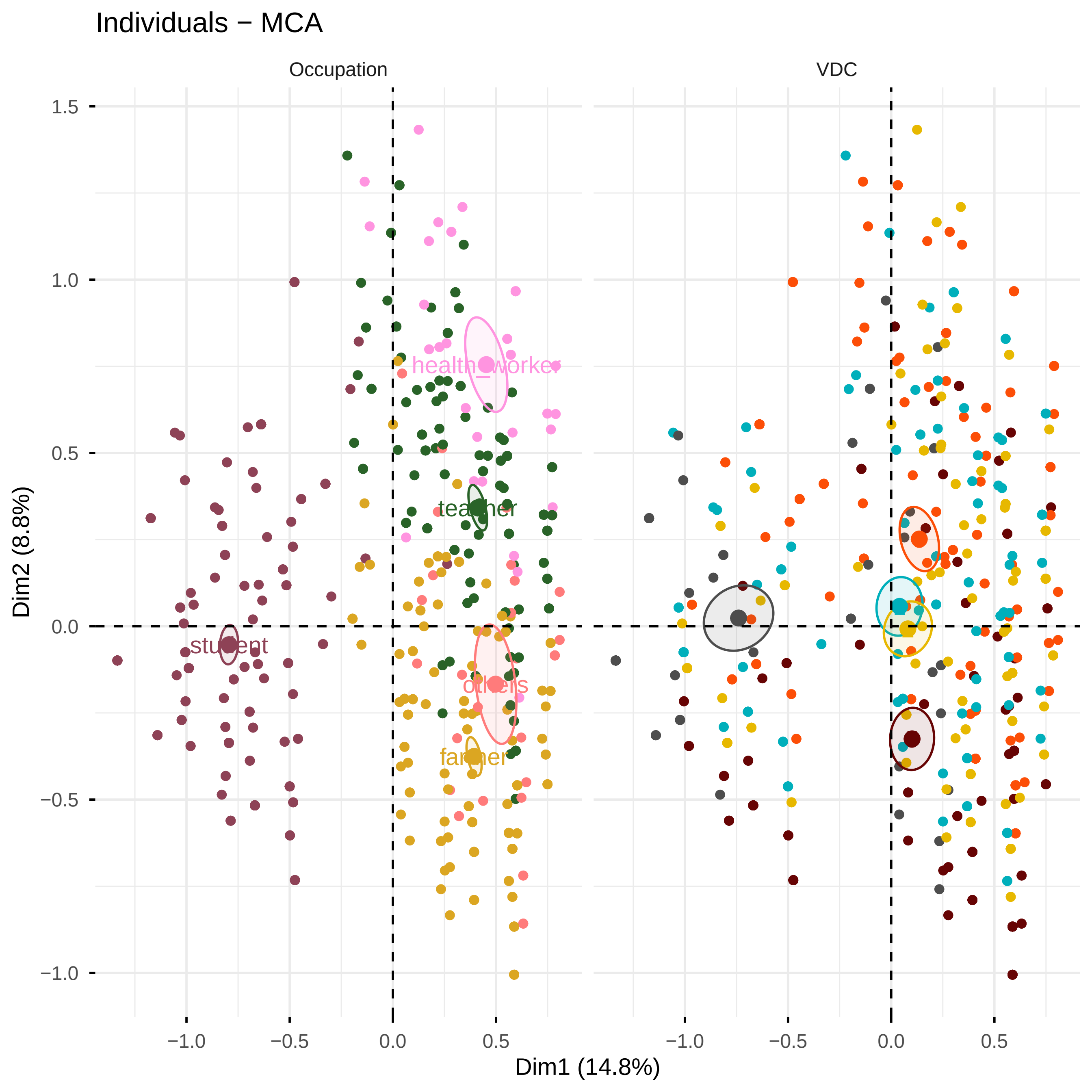

Individuals − MCA
Occupation
VDC
health_worker
teacher
student
others
farmer
Dim2 (8.8%)
Dim1 (14.8%)
1.5
1.0
0.5
0.0
−0.5
−1.0

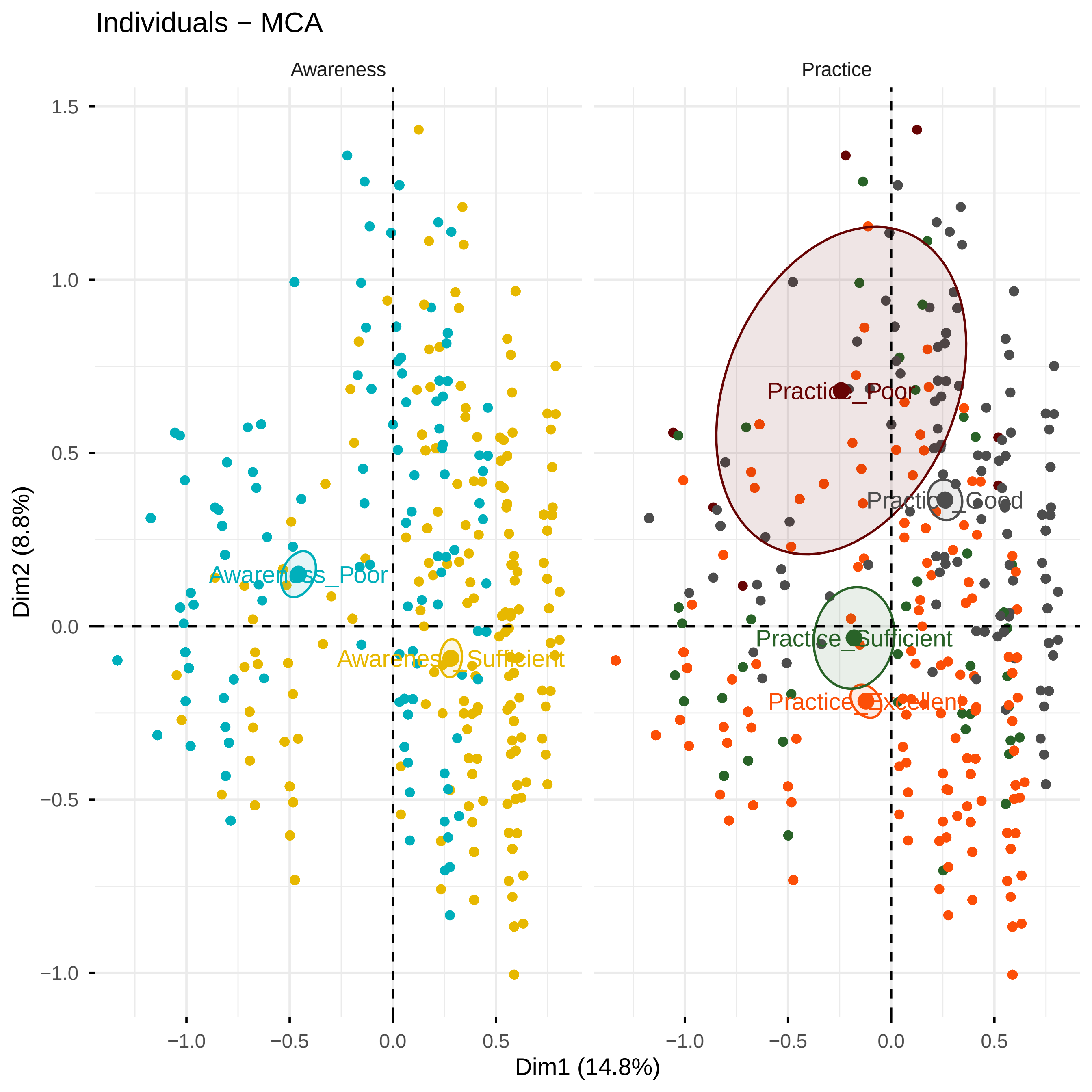

Individuals − MCA
Awareness
Practice
Awareness_Poor
Awareness_Sufficient
Practice_Poor
Practice_Good
Practice_Sufficient
Practice_Excellent
Dim1 (14.8%)
Dim2 (8.8%)
1.5
1.0
0.5
0.0
−0.5
−1.0
−1.0
−0.5
0.0
0.5

Figure S2h: Rabies MCA plot of variables

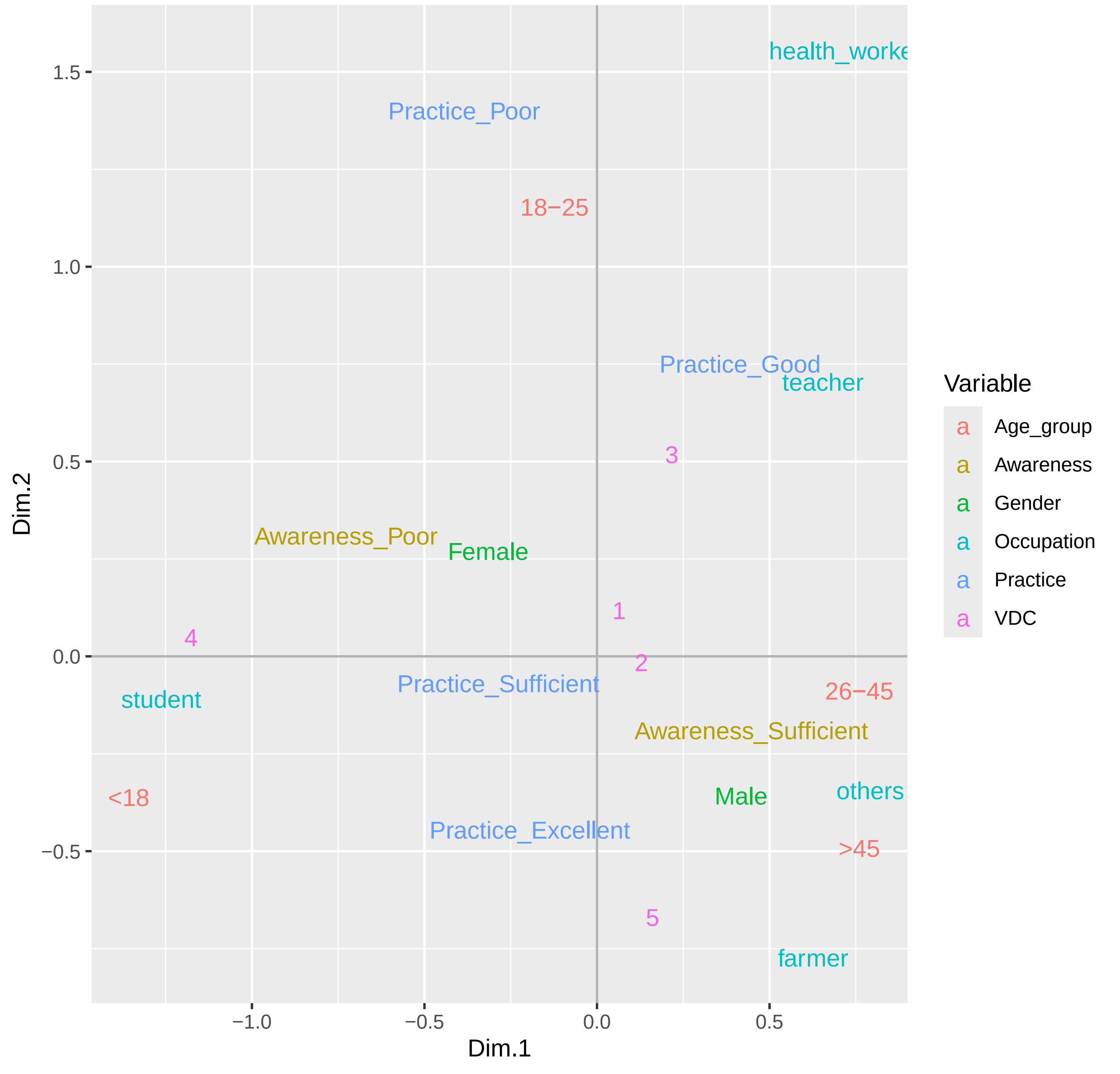

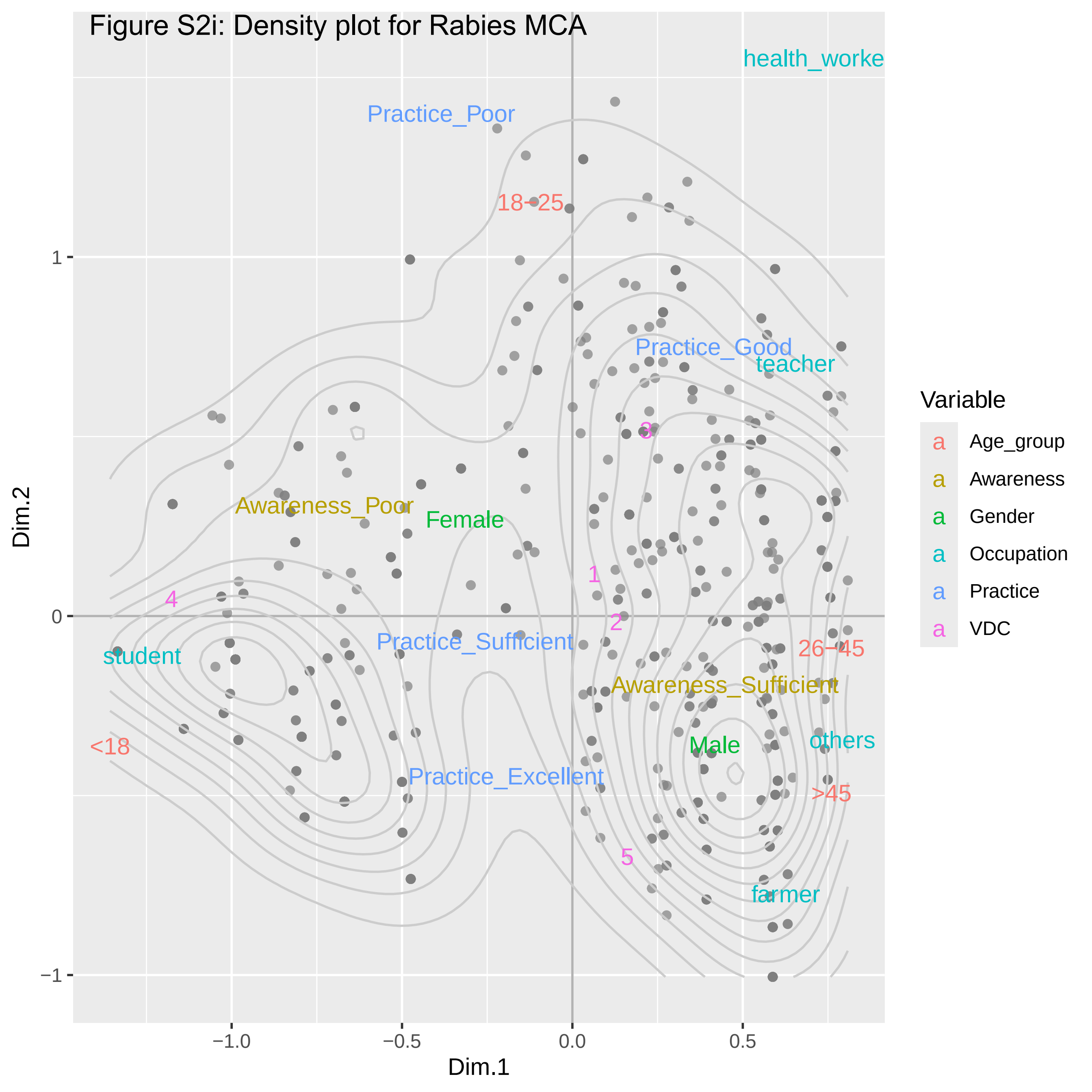

Figure S2i: Density plot for Rabies MCA
health_worke
Practice_Poor
18−25
Practice_Good
teacher
3
Awareness_Poor
Female
1
4
2
Practice_Sufficient
student
26−45
Awareness_Sufficient
<18
Male
others
Practice_Excellent
>45
5
farmer
1
0
−1
−1.0
−0.5
0.0
0.5
Dim.1
Dim.2
Variable
Age_group
Awareness
Gender
Occupation
Practice
VDC

Table S1: Univariable regression analysis results of tuberculosis awareness association with variables used in the study.

Variables	Estimate	Std. Error	z value	Pr(>|z|)
(Intercept)	-3.69	0.32	-11.54	<0.000
Age	0.05	0.01	6.34	<0.000
Age groups				
< 18(Intercept)	-19.57	743.87	-0.03	0.979
18-25	17.10	743.87	0.02	0.982
26-45	18.10	743.87	0.02	0.981
>45	18.37	743.87	0.02	0.980
Gender				
(Intercept)	-1.67	0.15	-10.92	<0.000
Female	-0.60	0.23	-2.65	0.008
VDC				
4 (Intercept)	-4.38	1.01	-4.36	<0.000
1	2.37	1.03	2.29	0.022
2	2.24	1.04	2.16	0.031
3	2.88	1.02	2.81	0.005
5	2.53	1.03	2.46	0.014
Occupation				
(Intercept)	0.65	0.34	1.91	0.056
farmer	-2.93	0.41	-7.07	<0.000
others	-1.71	0.53	-3.20	0.001
student	-20.22	673.44	-0.03	0.976
teacher	-2.15	0.39	-5.56	<0.000

Table S2: Univariable regression analysis results of rabies awareness association with variables used in the study.

Variables	Estimate	Std. Error	z value	Pr(>|z|)
(Intercept)	-0.98	0.20	-4.94	<0.000
Age	0.05	0.01	7.68	<0.000
Age groups				
< 18(Intercept)	-0.42	0.14	-2.95	0.003
18-25	0.49	0.22	2.22	0.026
26-45	1.70	0.20	8.29	<0.000
>45	1.53	0.25	6.15	<0.000
Gender				
(Intercept)	0.90	0.12	7.29	<0.000
Female	-0.69	0.16	-4.42	<0.000
VDC				
4 (Intercept)	-0.43	0.23	-1.87	0.061
1	0.89	0.28	3.20	0.001
2	0.90	0.28	3.26	0.001
3	0.69	0.27	2.50	0.013
5	1.79	0.30	5.97	<0.000
Occupation				
Health worker (Intercept)	1.67	0.44	3.76	0.000
farmer	-0.53	0.47	-1.12	0.261
others	-0.62	0.61	-1.02	0.307
student	-2.21	0.46	-4.77	<0.000
teacher	-0.71	0.47	-1.50	0.134
